# The human intelligence evolved from proximal *cis*‐regulatory saltations

**DOI:** 10.1002/qub2.88

**Published:** 2025-01-03

**Authors:** Xiaojie Li, Jianhui Shi, Lei M. Li

**Affiliations:** ^1^ Academy of Mathematics and Systems Science Chinese Academy of Sciences Beijing China; ^2^ School of Mathematical Sciences University of the Chinese Academy of Sciences Beijing China

**Keywords:** Alu element, gene regulation, human intelligence, saltation, serotonin

## Abstract

The divergence rate between the alignable genomes of humans and chimpanzees is as little as 1.23%. Their phenotypical difference was hypothesized to be accounted for by gene regulation. We construct the *cis*‐regulatory element frequency (CREF) matrix to represent the proximal regulatory sequences for each species. Each CREF matrix is further decomposed into dual eigen‐modules. By comparing the CREF modules of four existing hominid species, we examine their quantitative and qualitative changes along evolution. We identified two saltations: one between the 4th and 5th, the other between the 9th and 10th eigen‐levels. The cognition and intelligence unique to humans are thus found from the saltations at the molecular level. They include long‐term memory, cochlea/inner ear morphogenesis that enables the development of human language/music, social behavior that allows us to live together peacefully and to work collaboratively, and visual/observational/associative learning. Moreover, we found exploratory behavior crucial for humans’ creativity, the GABA‐B receptor activation that protects our neurons, and serotonin biosynthesis/signaling that regulates our happiness. We observed a remarkable increase in the number of motifs present on Alu elements on the 4th/9th motif‐eigenvectors. The cognition and intelligence unique to humans can, by and large, be identified using only the CREF profiles without any a priori. Although gradual evolution might be the only mode in the mutations of protein sequences, the evolution of gene regulation has both gradual and saltational modes, which could be explained by the framework of CREF eigen‐modules.

**Abbreviations**
CREF: *cis*‐regulatory element frequency
CREI: *cis*‐regulatory element incidence
CREs: *cis*‐regulatory elements
GABA: gamma‐aminobutyric acid
minFN: minimize false negative rate
minFP: minimize false positive rate
MPA: motifs present on Alu elements
PWM: position weight matrix
SVA: SINE‐R/VNTR/Alu
SVD: singular value decomposition
TFs: transcription factors
TSS: transcription start sites

## 1 INTRODUCTION

The availability of high‐quality genomes of major species makes it possible to reach a better answer to the problem “What genetic changes made us uniquely Human?”, one of the 25 most important questions as stated by *Science*, on its 125th anniversary. Clues to the above questions could be found in the context of humans’ closest relatives. The existing hominid species are from four genera: *Pongo* (the Bornean, Sumatran, and Tapanuli orangutan); *Gorilla* (the eastern and western gorilla); *Pan* (the chimpanzee and the bonobo); and *Homo*. Modern humans (*Homo sapiens*) are the only *Homo* species that survive. Among the various phenotypes of species, human intelligence is a drastic or jump change compared to all other apes. A jump change is generally referred to as saltation.

Comparative genomics of existing hominid species is a powerful tool to understand the evolution of humans. The pioneering work was carried out by King and Wilson. Surprisingly, when researchers examine the genomic regions of humans and chimpanzees that can be aligned, the divergence rate at the nucleotide level is as little as 1.23%. King and Wilson (1975) proposed that cues for biological differences might be in gene regulation [1].

Transcriptional regulation is a complex process that involves several components and processes such as topological association domains, chromatin remodeling, histone modification, and the interaction between *cis*‐elements and *trans*‐factors [2, 3, 4]. The *cis*‐*trans* regulation is the key step. The structures of transcription factor proteins are relatively conserved between closely related species such as between humans and chimpanzees, as indicated by the small difference in their DNA coding sequences. Changes in *cis*‐regulatory sequences constitute an essential part of the genetic basis for evolution and adaptation [5]. Although the claim has been supported by various case studies, a systematic model that can explain the dramatic phenotypical difference between humans and apes is still lacking.

Mathematically, DNA evolution has been modeled as a continuous‐time Markov chain. Examples include the JC69 model and the K81 model [6, 7]. These models are appropriate for single nucleotide mutations, and many successes have been achieved. On the other hand, the *cis*‐regulatory sequences are modified not only by single nucleotide mutations but also by other types such as short tandem repeats and insertions of transposons [8, 9, 10]. How to represent *cis*‐regulatory sequences in such a way that various types of mutations can be taken into account is a challenge at this time.

King and Wilson (1975) even proposed that a relatively small number of genetic changes in the system controlling the gene expressions may account for the major organismal differences between humans and chimpanzees [1]. A similar situation exists in physics and chemistry. That is, a small change in the controlling parameter of a particle system alters the interactions at the microlevel and results in a drastic change of a physical property at the macrolevel. This phenomenon is called a phase transition. Various mathematical models have been developed in the last century to explain phase transitions. It is interesting to know if these mathematical insights were applicable to the evolution of *cis*‐regulatory sequences.

In 2020, we made an initial effort to explain the phenotypical difference between humans and apes by the *cis*‐regulatory sequences [11]. We constructed a *cis*‐regulatory element frequency (CREF) matrix for each species. Such a CREF matrix was then decomposed into dual eigen‐modules that can be interpreted into biology through polarized gene‐ and *cis*‐motif‐eigenvectors. Then, we aligned and compared the CREF dual eigen‐modules across humans, chimpanzees, and orangutans. The top three and the 6th CREF modules are highly conserved. Surprisingly between the 4th and 5th levels, the CREF modules underwent a phase transition, which we refer to as saltation hereafter in this paper. This saltation led to a human‐specific 4th CREF module that regulates long‐term memory, cochlea development, social behavior, sympathetic nervous systems etc. The dual eigen‐analysis indicates that one key genetic driving force for the saltation of CREF modules is mutations relating to Alu which has over one million copies in the human genome.

In this article, other than humans and chimpanzees, we include bonobos and gorillas in the comparison of the CREF modules. The comparison goes beyond the 6th level till the 10th level. A rotation of eigenvectors between the 9th and 10th levels occurred. The transition led to a module that characterizes intelligence, yet somewhat different from that between the 4th and 5th levels. The article first described the CREF approach by adding some details, in particular, providing evidence on *cis*‐element multiplicities, and highlighting that module sensitivity is inversely proportional to the distance between adjacent eigenvalues. Then, the saltation between the 9th and 10th levels is reported and compared to that between the 4th and 5th levels, with an emphasis on human intelligence. One important aspect of intelligence is the learning ability of various types. In addition to Alus, we also consider the role of the transposon SVAs in the saltations.

## 2 RESULTS

**FIGURE 1:**
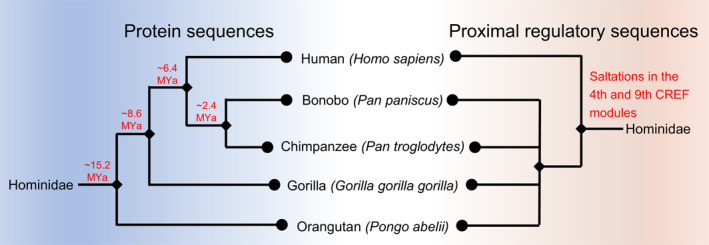
The phylogenetic relationship of humans and four apes based on protein sequences and the tree based on proximal regulatory sequences. The left is based on protein sequences and the divergence times are marked on the tree; the right is based on the 4th and 9th CREF modules obtained from proximal regulatory sequences and the position of the saltations of regulatory modules is marked on the tree.

The phylogenetic tree of humans, chimpanzees, bonobos, and gorillas based on protein sequences is shown on the left of Figure 1. It is obtained from fossils and molecular evidence. The estimation of divergence time requires the molecular clock, strict or relaxed [12]. In this article, we study the evolution of *cis*‐*trans* regulation in Hominidae without any presumption. The availability of high‐quality genomes and annotations of the four hominid species allows us to study the regulatory DNA sequences of all the genes by a systems biology approach. The tree based on proximal regulatory sequences is shown on the right of Figure 1.

### 2.1 *Cis*‐regulatory elements

*Cis*‐regulatory elements (CREs) are specific and short (typically about 6–15 bp in length) noncoding DNA sequences that regulate the transcription of neighboring genes. CREs can be found in regulatory regions such as promoters, enhancers, and silencers. In this report, the CREF approach is applied to the regulatory regions near the transcription start sites (TSS) of all annotated protein genes.

*Cis*‐elements can specifically be bound by transcription factors (TFs). The protein structures of TFs are rather conserved across closely related species. In contrast, more variations are found in the DNA *cis*‐elements across species [13]. Mutations related to *cis*‐elements must account for quite a portion of the evolution of the gene regulatory mechanism.

The motif of *cis*‐elements is commonly depicted by a position weight matrix (PWM), which represents the probability distribution of bases at each position. Some motif databases such as TRANSFAC, JASPAR, and HOMER are available along with their motif‐search software. Given a motif PWM, its occurrences on a DNA regulatory sequence are identified by a motif‐search program. In this study, we chose the TRANSFAC database [14] as before [11]. Using the motif‐search program MATCH associated with the TRANSFAC database [15], we performed a systematic search for motifs of *cis*‐elements that are potential binding sites of transcription factors. It is reported that with the minFP (minimize false positive) option, the MATCH identified only half of the sites that were used to derive the motif position weight matrices [16]. Therefore, we took the minFN (minimize false negative) option in the construction of CREF matrices.

### 2.2 *Cis*‐regulatory element frequency matrices

**FIGURE 2:**
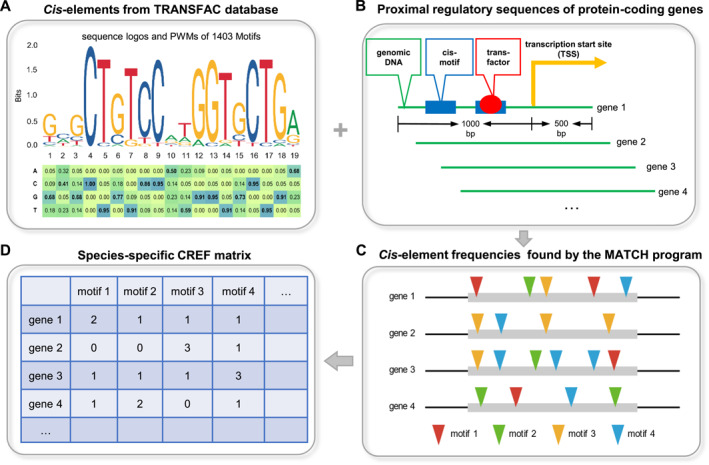
The flowchart of constructing a CREF matrix. (A) Choose a *cis*‐element database. In this study, we choose the TRANSFAC database in which 1403 motifs are represented by PWMs (position weight matrix) that can be visualized by sequence logos as shown. (B) Extract the proximal regulatory sequences of protein‐coding genes. The proximal regulatory sequence is defined to be the region between −1000 bp upstream and +500 bp downstream of the TSS (transcription start site). For a gene with multiple annotated transcripts, the start position of its most upstream transcript toward the 5' end is chosen as the TSS. (C) Search for potential *cis*‐*trans* binding sites in the proximal regulatory sequence. In this study, the search is carried out by the MATCH program, whose threshold option is taken to be minFN (minimize false negative). (D) Arrange the counts of the motifs into a species‐specific matrix with genes as rows and motifs as columns. The matrix is referred to as the *cis*‐regulatory element frequency (CREF) matrix on which this quantitative and systems biology study is based.

The central part of transcriptional regulation is its initialization, which occurs around the TSS. We focus on the proximal regulatory region at the 5' end, specifically, the region between 1000 bp upstream and 500 bp downstream TSS. Not only does such a region include the promoter of a gene but also the first intron in many cases. When a gene has multiple annotated transcripts, the start position of the most upstream transcript toward the 5' end is chosen as the TSS. The counts of *cis*‐elements of all protein‐coding genes are arranged into a matrix with genes as rows and motifs as columns. We termed it the *cis*‐regulatory element frequency (CREF) matrix of a species (Figure 2A–D). The CREF matrix is the basis of this quantitative and systems biology research.

### 2.3 Multiplicity of *cis*‐elements

**FIGURE 3:**
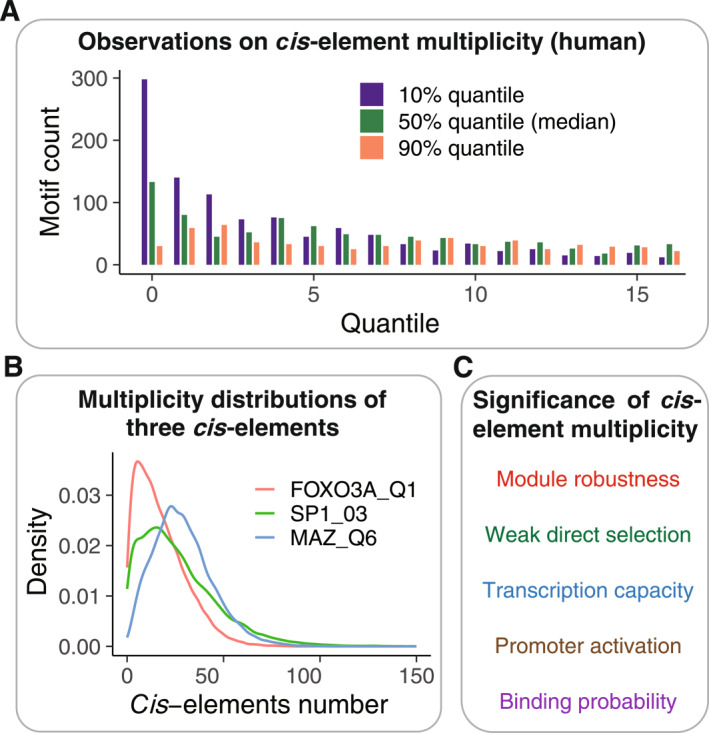
The multiplicity of *cis*‐element and its biological implications. (A) The distributions of quantiles (round to integers) of 1403 motifs across human protein genes. The 10%, 50%, and 90% quantiles of frequencies for each motif across all genes are computed. Their distributions of 1403 motifs are shown in purple, green, and orange, respectively. It is noted that the number of motifs whose frequency medians do not exceed 1 is less than 220. This indicates that most motifs have multiple occurrences in at least half of the gene regulatory regions. The relatively flat distribution of 90% quantiles between 4 and 16 (orange) indicates the strong *cis*‐binding capacity that varies in a wide range. (B) The distributions of multiplicities for the three *cis*‐element motifs: MAZ_Q6, SP1_03, and FOXO3A_Q1 across 19,354 genes in humans. Their modes are respectively 23, 16, and 5. They demonstrated different *cis*‐element frequency patterns across *cis*‐elements. (C) The biological implications of the *cis*‐element multiplicity. These implications were supported by experimental and computational evidence reported in the literature.

Cracking the regulation code by CREF is motivated by the observation that a *cis*‐element is usually found in clusters. To investigate the multiplicities of *cis*‐elements in genes’ proximal regulatory regions, we obtain their in silico distributions by the motif‐search program. Take the human genome for example: its CREF matrix includes 19,354 protein‐coding genes as rows and 1403 *cis*‐motifs as columns. Each motif has its specific distribution of occurrences across 19,354 genes. To summarize the 1403 distributions, we display their 10%, 50% (median), and 90% quantiles in counts in Figure 3A. Nearly 300 motifs are absent in 10% of genes, and 130 motifs are absent in 50% of genes. If a motif is present, its frequencies spread out, as seen from the distribution of medians (Figure 3A). The 90% quantiles, the top strongest binding signals, are quite uniform from 4 to 16, indicating that the *cis*‐element frequency is an informative measure of gene regulation from the view of information theory. Comparative analysis across species demonstrates that the pattern of *cis*‐element distributions is fairly conserved between humans and chimpanzees (Figure S1).

Three specific examples, MAZ_Q6, SP1_03, and FOXO3A_Q1, are shown in Figure 3B. The former two are GC‐richer, and their modes are respectively 23, 16, and 5. They demonstrated different *cis*‐element frequency patterns across *cis*‐elements.

The biological implications of the ubiquitous multiplicities of *cis*‐elements were important yet not fully understood. Some relevant progress and perspectives are given as follows (Figure 3C). First, it was argued that the multiplicity of *cis*‐elements can ensure the robustness of the regulatory module, and the weak direct selection provided by multiplicity plays an important role in organisms with large populations [17].

Second, the abundance of mRNA molecules proximal to the transcription site bears a rough proportionality to the quantity of bound transcription factors, which can be estimated by the number of binding sites [18]. Therefore, the frequency of the *cis*‐elements measures the transcription capacity. Third, a recent single‐cell research demonstrated that transcription factor binding quantities linearly contribute to promoter activation [19] and, in turn, control the gene expressions. These pieces of evidence indicate that multiple TFs bindings play a pivotal role in gene regulation.

Finally, it has been reported that the probability of TFs binding is proportional to the number of *cis*‐elements within a certain range [20]. The multiplicity of *cis*‐elements motivated us to construct the CREF matrix to represent the proximal regulatory sequences.

### 2.4 Sources of CREF variations

**FIGURE 4:**
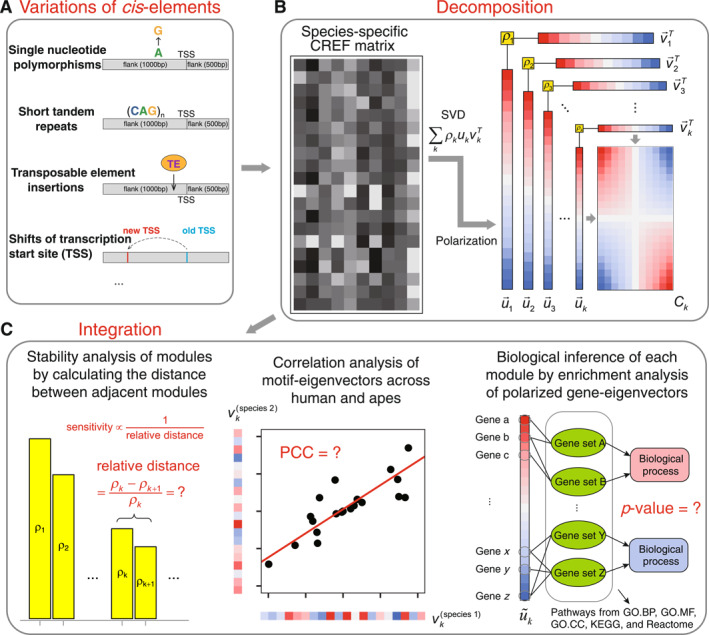
The scheme of the CREF dual eigen‐analysis. (A) The source of variations in *cis*‐regulatory elements. The single nucleotide polymorphisms, short tandem repeats, and insertions of transposon elements are genetic variations. The transcription start site (TSS) shifts that play functional and regulatory roles lead to variations in CREF matrices as well. (B) Decomposition of a CREF matrix. The species‐specific CREF matrix is stratified into multiple dual eigen‐modules through robust SVD (singular value decomposition) followed by polarization on both gene‐ and motif‐eigenvectors. Polarization means sorting loadings in descending order. The product of each polarized gene‐ and motif‐eigenvector shows a checkerboard pattern. (C) Integration of CREF modules. The integration mainly consists of three steps: stability analysis via relative distances between adjacent singular values, correlation analysis of motif eigenvectors, and statistical inference of molecular pathways and processes enriched at each module.

The variations in the CREF matrices across species primarily come from several sources (Figure 4A): single nucleotide polymorphisms (point mutations), short tandem repeats, insertions of transposable elements, shifts of the TSS, etc. It is reported that the proportion of repeat sequences such as transposable elements reaches 52.1% of the whole human genome [21]. We will show how transposons influence the CREF matrix with examples later.

### 2.5 Dual eigen‐analysis of CREF matrix

The dual eigen‐analysis consists of two steps: decomposition and integration (Figure 4B–C). In the decomposition step, we aim to embed the regulatory mechanism underlying a large‐scale CREF matrix into a structure of lower dimension. Thus, the standard dimension reduction tool, singular value decomposition (SVD) comes to the need.

We denote a CREF matrix by $\tilde{C}=\left({\tilde{c}}_{ij}\right)$, where i=1,...,g,j=1,...,m,g is the total number of protein‐coding genes, and m is the total number of *cis*‐motifs. To mitigate the influences of outliers and noise, we apply a robust version of the SVD method. That is, we take the decomposition $\tilde{C}=C+S$, where C is expected to capture the principal regulation information and S is a sparse matrix that represents the unexpected outliers and noise. We reconstruct C from $\tilde{C}$ by solving the following optimization problem:

$$\underset{C,S}{\min\;}\|C{\|}_{*}+\lambda \|S{\|}_{1}\;\mathrm{subject}\;\mathrm{to}\;\tilde{C}=C+S,$$

where ${\left\|\cdot \right\|}_{*}$ denotes the nuclear norm of a matrix, that is, the sum of its singular values, ${\left\|\cdot \right\|}_{1}$ denotes the sum of the absolute values of the matrix entries, and λ is a penalizing parameter. To solve the above optimization problem, we choose the inexact augmented Lagrange multipliers (IALM) algorithm [22]. Then, we consider the SVD of $C:C=\sum_{k=0}^{s}\rho_{k}u_{k}v_{k}^{T}$. Singular values are sorted from large to small, that is, $\rho_{0}>\rho_{1}>\rho_{2}>\text{⋯}>\rho_{s}$. The zero‐level eigen‐component corresponds to the baseline and is skipped hereafter.

Next, we polarize the gene‐eigenvector $u_{k}$ and motif‐eigenvector $v_{k}$ by sorting their loadings. The sorted eigenvectors ${\vec{u}}_{k}$ and ${\vec{v}}_{k}$ are respectively referred to as the polarized gene‐ and motif‐eigenvectors. Most loadings of the eigenvectors are close to zeros, and only the genes and motifs near the two ends of ${\vec{u}}_{k}$ and ${\vec{v}}_{k}$ need further attention. At each level, the pair of polarized gene‐ and motif‐eigenvectors together with the singular value constitute a dual eigen‐module. Intra‐species comparisons involve different levels, while inter‐species comparisons focus on the same level.

The integration step has three components: (i) evaluate the stability of each dual eigen‐module; (ii) compare motif‐eigenvectors $\left\{v_{k}\right\}$ across species by Pearson correlation coefficients; and (iii) infer the biological processes enriched at the poles of polarized gene‐eigenvectors $\left\{{\vec{u}}_{k}\right\}$ (Figure 4C).

### 2.6 Stability of CREF eigen‐modules

**FIGURE 5:**
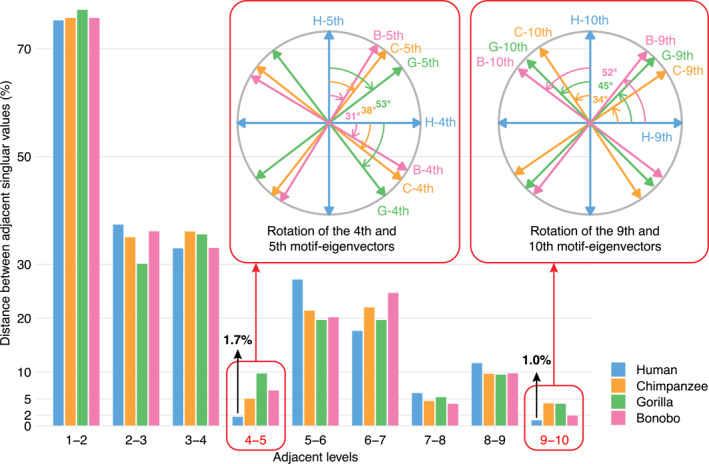
The rotations of the 4th/5th and 9th/10th motif‐eigenvectors from humans to the three apes and the relative distances between adjacent modules up to the 10th level. Bottom: Compared to those in the three apes, the relative distances are as low as 1.7% between the 4th and 5th levels and 1.0% between the 9th and 10th levels in humans. Meanwhile, they are notably lower than those between other adjacent levels. Large relative distances between adjacent singular values imply conserved modules across species. Top: All three apes exhibit large rotations ranging from 31° to 53° in the 4th/5th motif‐eigenvectors and ranging from 34° to 52° in the 9th/10th ones. Small relative distances and large rotations both imply saltations in the human 4th/5th and 9th/10th modules.

We quantitatively measure the relative distance between adjacent modules by the following formula: $d_{k}=\left(\rho_{k}-\rho_{k+1}\right)/\rho_{k}$, where $\rho_{k}$ represents the k‐th singular value. The relative distances are as low as 1.7% between the 4th and 5th levels and 1.0% between the 9th and 10th levels in humans. They are notably lower than those in the three apes (Figure 5). The relative distances between other adjacent levels are much larger, particularly in humans.

The magnitude of the relative distance measures the stability of the dual eigen‐module. Following the previous notation, by the nature of SVD, we have $C^{T}C=\sum_{k=0}^{s}\rho_{k}^{2}v_{k}v_{k}^{T}$. When $C^{T}C$ is added by a symmetric perturbation E, the perturbed eigenvectors of two adjacent eigen‐modules have the following expansions under certain regular conditions:

$$\left\{\begin{array}{l} {\hat{v}}_{k}=v_{k}+\frac{v_{k+1}^{T}Ev_{k}}{\rho_{k}-\rho_{k+1}}v_{k+1}+\sum_{j\neq k,k+1}\frac{v_{j}^{T}Ev_{k}}{\rho_{k}-\rho_{j}}+O\left({\left\|E\right\|}_{2}^{2}\right)\\ {\hat{v}}_{k+1}=v_{k+1}-\frac{v_{k}^{T}Ev_{k+1}}{\rho_{k}-\rho_{k+1}}v_{k}+\sum_{j\neq k,k+1}\frac{v_{j}^{T}Ev_{k+1}}{\rho_{k+1}-\rho_{j}}+O\left({\left\|E\right\|}_{2}^{2}\right) \end{array}\right.,$$

where ${\hat{v}}_{k}$ and ${\hat{v}}_{k+1}$ represent the k‐th and (k+1)‐th perturbed eigenvectors, respectively, ${\left\|\cdot \right\|}_{2}$ denotes the spectral norm of a matrix, and O indicates the asymptotic upper bound. The above expansion is the first‐order approximation, which is usually given in a matrix form [23]. The derivation from the matrix form can be found in Dr. Liang Li’s thesis [24]. The first‐order expansion can be derived directly [25]. As indicated by the expansion, the sensitivity of the k‐th and (k+1)‐th eigen‐modules is approximately inversely proportional to the distance between their respective eigenvalues. After we normalize the distance by the local scale parameter $\rho_{k}$, it becomes

$$\mathrm{sensitivity}\propto d_{k}^{-1}.$$

Mathematically, as two singular values become identical, their corresponding 2‐D dual eigen‐spaces become degenerate. In either the degenerate motif or gene eigen‐space, any vector is an eigenvector of the same singular value. When the adjacent singular values are close, both the motif‐ and gene‐eigenvectors are sensitive to perturbation. In the case of humans, the 4th and 5th modules are not stable, and neither do the 9th and 10th ones. Consequently, perturbation to the CREF matrix would lead to qualitative changes rather than quantitative changes between these two pair modules. In other words, saltations may occur.

### 2.7 Saltations between the 4th and 5th as well as between the 9th and 10th levels

Along the motif‐eigenvectors, we are able to use the Pearson correlation coefficient to measure their similarities across species since they have the same dimensions. Based on the correlation coefficient, each eigen‐module is judged to be conserved or not. A larger correlation coefficient indicates a more conserved module. In this study, we conduct the comparison from the first to the 10th level.

**FIGURE 6:**
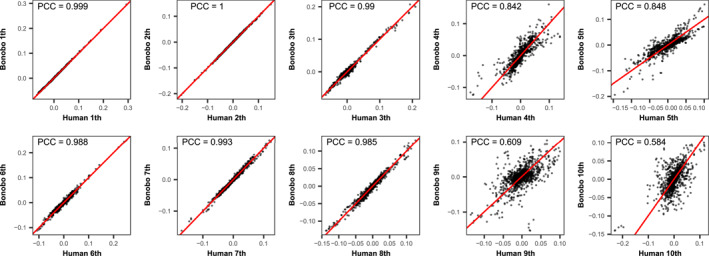
The scatter plots of humans’ motif‐eigenvector loadings versus bonobos’ from levels 1 to 10. The straight lines are fitted by Deming regression. Unlike the simple ordinary linear regression, it accounts for errors in observations on both the *x*‐ and the *y*‐axis. The Pearson correlation coefficients at levels 4, 5, 9, and 10 are, respectively, 0.842, 0.848, 0.609, and 0.584, showing certain divergences. In contrast, the modules at other levels are highly conserved with Pearson correlation coefficients all greater than 0.985. This indicates striking saltations occur between the 4th and 5th levels and between the 9th and 10th levels. Scatter plots of other pair species can be found in Figures S2–S6.

Among humans, bonobos, chimpanzees, and gorillas, the correlation coefficients of the top three and the 6th motif‐eigenvectors all exceed 0.99, indicating that these modules are highly conserved (Figure 6, S2−S6). While the modules of the 7th and 8th are conserved among humans, bonobos, and chimpanzees, as indicated by the correlation coefficients that exceed 0.98 (Figures 6, S2, S5), they showed certain divergence in gorillas, as the correlation coefficients drop into the interval [0.82, 0.88] (Figures S3, S4, S6).

A striking transition occurs between the 4th and 5th levels. The correlation coefficients between bonobos and chimpanzees are as high as 0.99/0.99 (Figure S2); those between gorillas and chimpanzees are 0.97/0.96 (Figure S3); and those between gorillas and bonobos are 0.92/0.92 (Figure S4). In contrast, those between humans and bonobos, chimpanzees, and gorillas are respectively 0.84/0.85, 0.79/0.79, and 0.61/0.60 (Figures 6, S5, S6). Therefore, a significant transition occurred between the 4th and 5th levels in humans but not in the three apes.

A similar transition was observed between the 9th and 10th levels. The correlation coefficients between bonobos and chimpanzees are 0.93/0.93 (Figure S2); those between gorillas and chimpanzees are 0.98/0.97 (Figure S3); and those between gorillas and bonobos are 0.98/0.93 (Figure S4). In contrast, those between humans and bonobos, chimpanzees as well as gorillas drop down to 0.61/0.58, 0.82/0.81, and 0.70/0.68, respectively (Figures 6, S5, S6).

Next, from another angle, we consider the transition between the 4th and 5th and that between the 9th and 10th levels. By projecting the three apes’ motif‐eigenvectors onto the human ones, it can be seen that the 4th motif‐eigenvectors of bonobos, chimpanzees, and gorillas rotate by 31°, 38°, and 53° from that of humans, and the 9th ones rotate by 52°, 34°, and 45°, respectively (Figure 5). It is noted that at levels 4 and 5, bonobos are slightly closer to humans than chimpanzees, while at levels 9 and 10, the reverse is true.

### 2.8 Intelligence and cognition processes enriched at the 4th CREF eigen‐modules

Along the gene‐eigenvectors, we performed the enrichment analysis via the rank‐based Wilcoxon test [26]. Those intelligence‐related biological processes whose genes rank significantly higher than others are identified (Table S1). The key biological processes enriched at the top 6 levels have been previously documented. In particular, the 4th human gene‐eigenvector resulted from the saltation led to specific cognition and intelligence module including long‐term memory, cochlea morphogenesis, social behavior, and sympathetic nervous system development.

**FIGURE 7:**
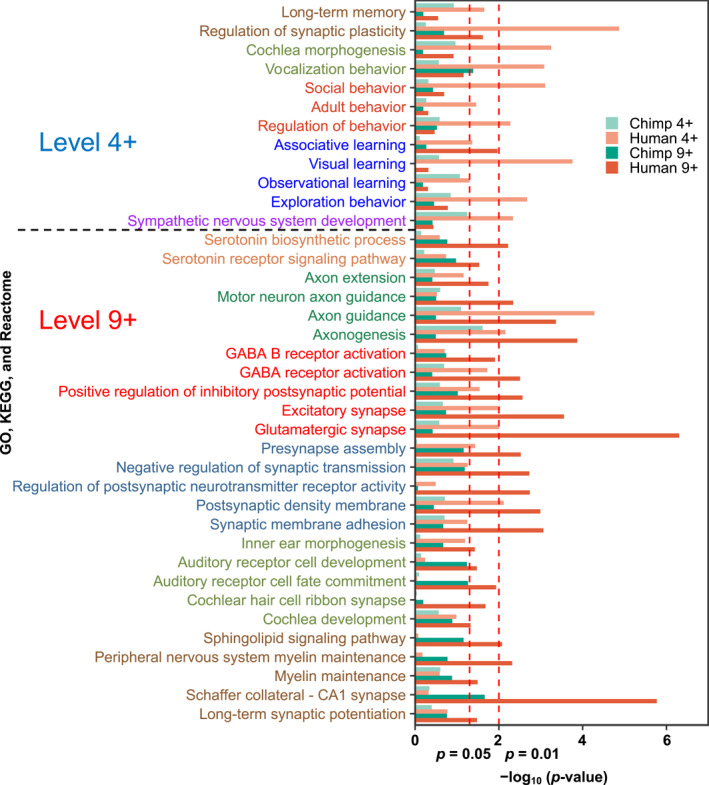
Intelligence‐related biological processes significantly enriched near the positive poles of the 4th and 9th polarized gene‐eigenvectors of humans but not so along those of chimpanzees. The enrichment analysis is performed via the rank‐based Wilcoxon test [72]. The relevant gene subsets are collected from the three knowledge bases: Gene Ontology [73, 74], KEGG [75, 76], and Reactome [77]. The significances in terms of log(*p*‐values) at the human 4th and 9th, chimpanzee 4th and 9th eigenvectors are represented by the light orange and dark orange, light green and dark green bars, respectively. The left red‐dashed line marks the *p*‐value 0.05 and the right one marks the *p*‐value 0.01. The enriched gene subsets are grouped according to their biological meanings in different colors.

In this article, we further reported the learning abilities enriched at the human 4th CREF module (the upper part of Figure 7). They include visual learning and observational learning that rely on impressions of sensation. Also statistically significant is the more sophisticated associative learning that establishes new responses as a result of the repeated presentation of paired stimuli. According to the classification by David Hume, an association could be due to resemblance, contiguity in time and place, or causation that is beyond our senses [27].

In the 4th module, humans demonstrate significant exploration behavior. Exploration behavior is motivated by curiosity, and its outcome can lead to punishment or rewards. Memory, associative learning, and the brain’s reward system are highly interconnected with exploration behavior [28].

### 2.9 Intelligence and cognition processes enriched at the 9th CREF eigen‐modules

The lower part of Figure 7 showed the pathways significantly enriched at the positive pole of the human 9th level. They include associative learning as well. Pathways such as axonogenesis and axon guidance are all significantly enriched and so are they at the 4th level. Multiple synapsis‐related processes are enriched. Examples include glutamatergic synapse, presynapse assembly, synaptic membrane adhesion, etc.

Of note, GABA‐B synaptic receptors are significantly enriched at the 9th level, whereas GABA‐A synaptic receptors are enriched at the 4th level. The GABA receptors respond to the inhibitory neurotransmitter gamma‐aminobutyric acid (GABA) in the mature vertebrate central nervous system. GABA‐A receptors are ligand‐gated ion channels, whereas GABA‐B receptors are G protein‐coupled receptors. The reversal potential of the GABA‐B mediated inhibitory postsynaptic potential is −100 mV, much higher than that mediated by GABA‐A. Indeed, the enrichment *p*‐values of positive regulation of inhibitory postsynaptic potential are 0.096 in chimpanzees and 0.0027 in humans.

Surprisingly, while dopamine‐related pathways are not differentially enriched between human and chimpanzee 4th/9th modules, key serotonin‐related pathways stand out in the human 9th module. They include serotonin biosynthetic process, serotonin receptor signaling pathway, and serotonin‐gated cation‐selective channel activity. Serotonin is a monoamine neurotransmitter that regulates various physiological activities such as behavior, mood, cognition, reward, memory, and gastrointestinal homeostasis [29]. It is no wonder that serotonin neurons and receptors are targets for a wide variety of therapeutic drugs.

Along the line of long‐term memory, myelin maintenance is enriched at the human 9th module. The most significant process is “Schaffer collateral–CA1 synapse”. Schaffer collaterals project to area CA1 of the hippocampus, which stores long‐term memories through synaptic plasticity. Consistently, “long‐term synaptic potentiation” is enriched in this module of humans.

The enhanced hearing ability of humans is shown by multiple biological processes such as inner ear morphogenesis, cochlea development, cochlear hair cell ribbon synapse, auditory receptor cell development, auditory receptor cell fate commitment, and auditory receptor cell stereocilium organization.

The genes in these enriched pathways that ranked in the top 1500 of the positive poles of the human 4th and 9th gene‐eigenvectors are listed in Table S2 and Table S3. From them, we picked the top 20 genes if they ranked lower than 1500 in chimpanzees or they belonged to multiple processes in both 4th and 9th gene‐eigenvectors (Tables 1 and 2).

**TABLE 1:** Key genes in the enriched gene sets relating to intelligence, ranking in the top 1500 of the human polarized gene‐eigenvector at level 4.

Gene name	Rank	Biological processes	Process number
MBD5	56	Regulation of behavior	1
GRP	78	Social behavior	1
SHANK1	91	Long‐term memory; associative learning; adult behavior; social behavior; vocalization behavior; excitatory synapse	6
SHANK3	100	Adult behavior; social behavior; vocalization behavior	3
ADCY6	129	GABA receptor activation	1
NTF4	177	Long‐term memory	1
SLC6A1	195	Associative learning	1
ADGRB1	215	Axonogenesis; regulation of synaptic plasticity	2
HPN	250	Cochlea morphogenesis	1
ATXN1	277	Social behavior	1
ATP2B4	285	Glutamatergic synapse	1
NRXN2	309	Adult behavior; social behavior; vocalization behavior	3
NLGN2	345	Social behavior; positive regulation of inhibitory postsynaptic potential; presynapse assembly; excitatory synapse	4
NF1	452	Visual learning; sympathetic nervous system development; observational learning	3
FZD2	520	Cochlea morphogenesis	1
RIMS3	597	Regulation of synaptic plasticity	1
NRXN1	924	Adult behavior; social behavior; vocalization behavior	3
KCNQ1	971	Social behavior	1
APOE	1105	Long‐term memory; glutamatergic synapse	2
GRIN2D	1479	Regulation of synaptic plasticity; postsynaptic density membrane	2

*Note*: Some genes are in multiple processes. The details and number of biological processes are shown in the right two columns.

**TABLE 2:** Key genes in the enriched gene sets relating to intelligence, ranking in the top 1500 of the human polarized gene‐eigenvector at level 9.

Gene name	Rank	Biological processes	Process number
PCDH17	22	Negative regulation of synaptic transmission; synaptic membrane adhesion	2
ADORA1	45	Sphingolipid signaling pathway	1
PAFAH1B1	74	Auditory receptor cell development; cochlea development	2
GNG8	113	GABA receptor activation; GABA B receptor activation	2
DCHS1	142	Cochlea development	1
LRRC4	161	Synaptic membrane adhesion; excitatory synapse; Schaffer collateral ‐ CA1 synapse	3
FYN	205	Schaffer collateral ‐ CA1 synapse; sphingolipid signaling pathway	2
GABRR1	219	GABA receptor activation	1
ADCY1	278	Axonogenesis; Schaffer collateral ‐ CA1 synapse; postsynaptic density membrane; glutamatergic synapse; GABA receptor activation; GABA B receptor activation	6
RIMS4	364	Regulation of synaptic plasticity	1
GABRR2	450	GABA receptor activation	1
SOBP	570	Inner ear morphogenesis; cochlea development	2
CACNG2	586	Schaffer collateral—CA1 synapse; postsynaptic density membrane	2
ADCY9	614	GABA receptor activation; GABA B receptor activation	2
GIPC1	625	Regulation of synaptic plasticity; Schaffer collateral ‐ CA1 synapse	2
DRD2	775	Axonogenesis; associative learning	2
TPH2	976	Serotonin biosynthetic process; serotonin and melatonin biosynthesis	2
HTR3B	1357	Serotonin receptor signaling pathway; serotonin‐activated cation‐selective channel complex; serotonin‐gated cation‐selective channel activity	3
AGT	1417	Associative learning	1
GABBR2	1452	GABA receptor activation; GABA B receptor activation	2

*Note*: Some genes are in multiple processes. The details and number of biological processes are shown in the right two columns.

### 2.10 Cognition‐related genes stand around the positive pole of the human 4th and 9th gene‐eigenvectors

Complementary to enrichment analysis that relies on the available gene subsets, we directly examine the genes that ranked high at the 4th and 9th levels. We selected the genes that ranked in the top 200 of the positive poles of the human 4th and 9th gene‐eigenvectors but outside the top 200 of the three apes (Table S4). Some of them are not in the gene subsets shown in Figure 7 and Table S1. As reported in the literature, many of the genes are involved in the cognition, mood, psychiatric disorders, and brain development of humans.

We first focus on the positive pole of the 4th level. DNMT3L is related to cognitive decline [30]. NAE1 has differential expression in the hippocampus [31]. SHANK1 is pivotal for the cognition and synaptic structure and is expressed in glial cells [32]. ZBTB45 is involved in glial differentiation of oligodendrocyte progenitor and neural cells [33].

Next, our attention turns to the 9th level. STX3 is involved in human long‐term memory [34]. IFNAR1 is the receptor of type I interferon, important for memory and cognition [35]. N4BP1 is involved in neural stem cell differentiation [36]. MED15 is significantly expressed in the nervous system [37]. KLHL22 is crucial for maintaining and differentiating the precursor pool [38]. PLOD2 is correlated with brain arteriovenous malformations [39]. VRK2 is related to schizophrenia (SCZ) and major depressive disorder (MDD) [40]. B4GALT5 is involved in neuronal generation and myelin formation [41].

### 2.11 Human intelligence regulators stand at the positive pole of the 4th and 9th motif‐eigenvectors

**FIGURE 8:**
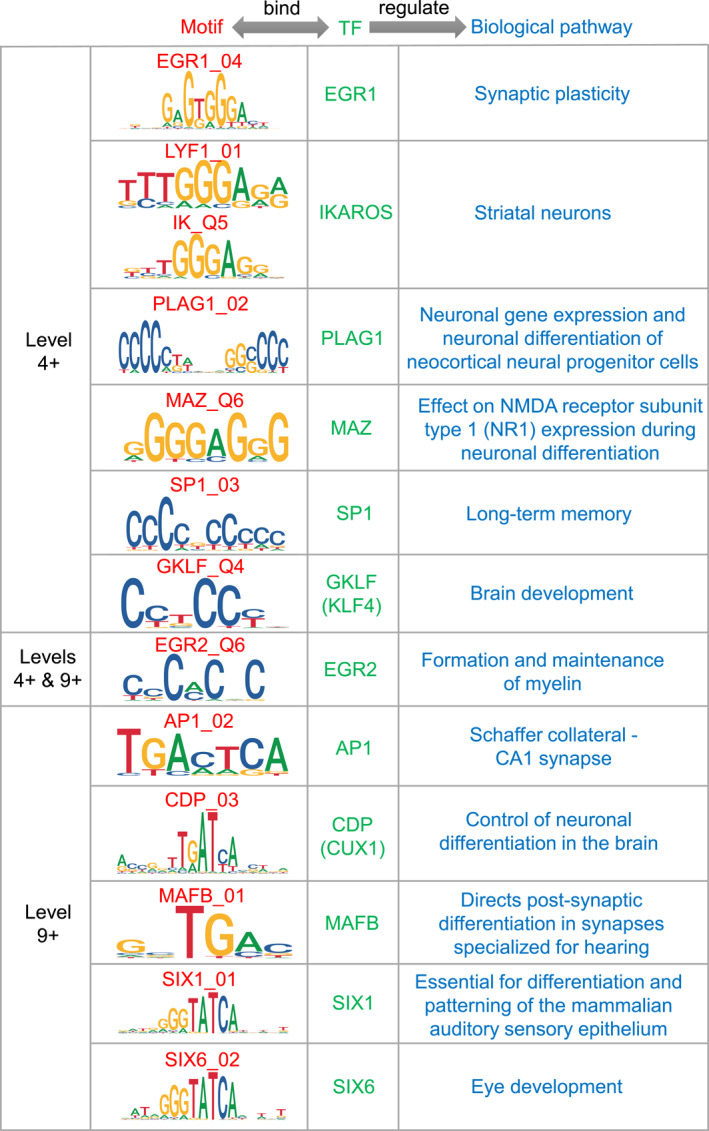
High‐ranking motifs standing at the positive poles of the 4th and 9th motif‐eigenvectors of humans but do not appear at those of the three apes. The dual eigen‐analysis automatically unveils the regulator–target gene relationship by decomposing the binding capacity profile. The second column displays the top‐ranked *cis*‐elements, whereas the third one displays their respective binding transcription factors. The human intelligence‐related biological processes comprised of the genes that are regulated by those in the second and third columns are shown in the last column. The regulator‐target annotations were compiled based on the functional genomic data reported in the literature. The module levels to which these regulatory relationships belong are shown in the first column.

The dual eigen‐analysis automatically unveils the regulator–target gene relationship by decomposing the binding capacity profile. With a close look at the positive pole of the human 4th and 9th motif‐eigenvectors, we find a list of high‐ranking *cis*‐motifs (Figure 8). The binding transcription factors of these motifs are reported to be involved in the regulation of several biological processes related to human intelligence.

We start with the positive pole of the 4th level. EGR1 was recognized as a marker of neuronal plasticity and plays a crucial role in the maintenance of synaptic plasticity [42]. It has a binding element EGR1_04 with a ranking of 36 in humans. SP1 and MAZ both are important regulators of long‐term memory. They were reported to be involved in mediating the enhancement of NMDA receptor subunit type 1 promoter activity during neuronal differentiation [43]. Their binding elements SP1_03 and MAZ_Q6 hold rankings of 56 and 30 in humans, respectively.

IKAROS (IK) was reported to play a role in regulating early temporal fates in the cerebral cortex [44]. It is also involved in the regulation of striatum neuron development through its DNA binding [45]. IKAROS has two binding *cis*‐elements LYF1_01 and IK_Q5 with rankings of 62 and 31 in humans, respectively. PLAG1 was shown to be involved in the regulation of neuronal gene expression and neuronal differentiation in neural progenitor cells of the developing mouse neocortex [46]. It serves as a determinant of neurogenic potential in neocortical neural progenitor cells [46]. PLAG1 has a binding element PLAG1_02 ranking 59 in humans. KLF4 was identified as a regulator of neurogenesis, neuronal differentiation, and neuronal migration during the formation of the cerebral cortex [47]. Its dysregulation causes hydrocephalus in postnatal mouse brains [48]. KLF4 has a binding element GKLF_02 ranking 28 in humans.

EGR2 (KROX20) is widely regarded as one of the cornerstones and central regulators of peripheral myelination [49]. EGR2_Q6, the binding element of EGR2, ranks 38 at the positive pole of the 4th level in humans. Surprisingly, it also stands at the positive pole of the 9th level in humans with a ranking of 68.

Next, our attention turns to the positive pole of the 9th level. AP1 is a marker of neuronal activation [42]. It was found to participate in regulating hippocampal CA1 areas [42]. AP1 has a binding element AP1_02 ranking 99 in humans.

CDP (CUX1) plays a critical role in dendritogenesis. It controls the number and maturation of dendritic spines [50]. One of its binding elements, CDP_03, ranks 30 in the human 9th level.

MAFB is responsible for driving the formation of auditory ribbon synapses, specialized structures for efficient transmission from cochlea hair cells to spiral ganglion neurons [51]. MAFB has a binding element MAFB_01 ranking 97 in humans. SIX1 plays an essential role in hair cell differentiation and the formation of the organ of Corti in the mammalian cochlea [52]. SIX1_01, the binding element of SIX1, holds a ranking of 7 in humans.

SIX6 (OPTX2) is a member of eye field transcription factors, which are demonstrated to be important in vertebrate eye formation [53]. SIX6 has a binding element SIX6_02 at the positive pole of the human 9th motif‐eigenvector with a rank of 10.

We note that almost all of these human high‐ranking motifs do not appear at the positive pole of the 4th and 9th motif‐eigenvectors of the three apes (rank out of the top 100). These *cis*‐element results are in line with their regulatory targets observed along gene‐eigenvectors. It is also noted that these regulator‐target annotations were compiled based on the functional genomic data reported in the literature.

### 2.12 Motifs present on Alu elements are key factors driving the transitions

Mutations related to Alu have been reported to be one of the key genetic driving forces for the Hominidae saltation between the 4th and 5th modules [54]. The relationship was found primarily through the motifs present on Alu elements (MPA). The number of MPAs at the pole of the 4th motif‐eigenvector increases substantially from chimpanzee to human [11]. To explore the more complete impact of MPAs on the gene regulation of human intelligence, we further compare their relative changes up to the 10th level for the three apes and humans.

**FIGURE 9:**
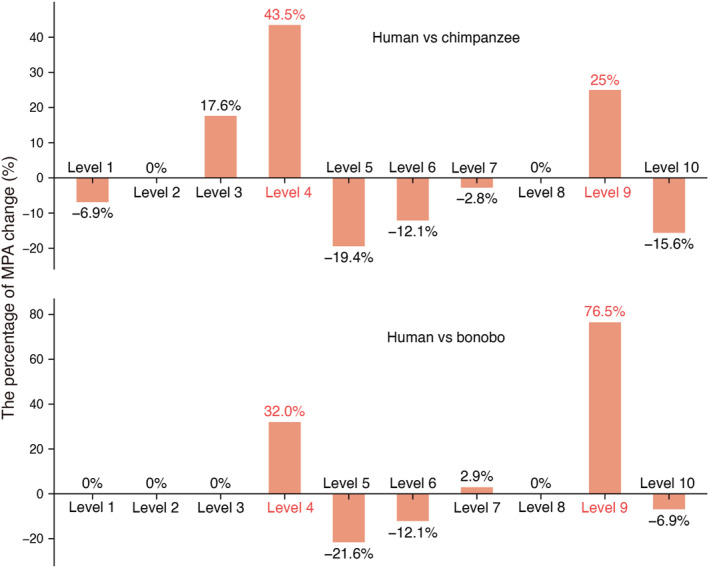
The relative change of MPAs (motifs present on Alu elements) in percentages at each level from two apes to human. Alu consensus sequences were obtained from the Repbase database [78]. They cover three major Alu subfamilies in primates: AluJ, AluY, and AluS. 200 MPAs were identified by the MATCH program with the threshold option taken to be minFN (minimize false negative rate). With reference to either chimpanzees or bonobos, the number of MPAs increases most significantly at the 4th and 9th levels in humans, indicating they are one driving force of the saltations. It is noted that in terms of MPA, bonobo is less different from human from level 1–4, while chimpanzee is less different from human at level 9.

Specifically, we calculate the relative change of MPAs from an ape to human in percentages at each level. With reference to chimpanzees, the number of MPAs in humans increases most significantly at level 4 by 43.5%, followed by 25% at level 9. With reference to bonobos, the number of MPAs increases most significantly at level 9 by 76.5%, followed by 32% at the 4th level (Figure 9). The same patterns are observed in gorillas (Figure S7). The substantial increase of MPAs at levels 4 and 9 implies that mutations related to Alu elements are likely to play an important role in the evolution of human intelligence.

### 2.13 Gene with human‐specific Alu insertion into its proximal regulatory region

Based on the aforementioned findings, the MPA frequency is a key factor in the saltation of human dual eigen‐modules [11]. Three mechanisms that Alu elements change the MPA frequencies were explained and exemplified in an earlier report [11]. Next, we list some genes that on the one hand rank high on either the 4th or 9th level and on the other hand have Alu insertions into their proximal regulatory regions. Specifically, at the 4th level, we found the gene BSG; at the 9th level, we identified five genes MGAT4C, OR11H2, CEBPG, UBE2T, and VDAC3 with human‐specific Alu insertions (Table 3). All of these genes rank in the top 1000 at the positive pole of human gene‐eigenvector but do not so of the other three apes. Notably, none of these genes are located on human chromosome 2, while two of them are positioned on chromosome 19. The human chromosome 19 has the characteristics of high gene density, high GC content, and high rearrangement rate [55].

**TABLE 3:** Genes with human‐specific Alu or SVA insertion at levels 4 and 9.

	Gene name	Chr	Alu/SVA name	Description	Rank
Level 4+	BSG	19	AluSg7	Dendrite self‐avoidance	45
Level 4+ & 9+	RTP3	3	SVA_F	Promote functional cell surface expression of the bitter taste receptors TAS2R16 and TAS2R43	954 & 331
Level 9+	MGAT4C	12	AluY	Related to cognition function, from GWAS	116
	OR11H2	14	AluYb8	Olfactory receptor family 11 subfamily H member 2	755
	CEBPG	19	AluYa5	Form stable heterodimers with CEBPB, a key regulator of long‐term memory	841
	UBE2T	1	AluYa5	Down‐regulation of expression in MEF2 signaling, playing a pivotal role in neuronal survival, differentiation, and synaptic plasticity	867
	VDAC3	8	AluYh3	Involved in synaptic transmission and learning	960
	RNASE10	14	SVA_D	Inactive ribonuclease‐like protein 10	73
	HYAL1	3	SVA_D	Related to myelination	265
	ERBB3	12	SVA_F	Involved in nervous system‐related pathways	849

*Note*: The table lists the gene names, the chromosomes where they are located, the types of inserted transposable elements, the short descriptions of genes, and their ranks in the polarized gene‐eigenvector.

Numerous studies revealed significant connections between these genes and human intelligence. A noteworthy instance is the BSG gene. Within the proximal regulatory region of a BSG’s transcript, we observed a human‐specific Alu insertion. The insertion element is AluSg7, which belongs to a relatively old Alu subfamily. Interestingly, the start site of this transcript shifted about 1300 bases toward 5' upstream according to the alignment on the UCSC browser (Figure S8). The BSG gene is related to dendrite self‐avoidance, a key factor in neural network construction and memory formation. A recent report showed that synaptic connections in the human brain are directed and acyclic topologies [56]. Self‐avoidance is likely to mediate the formation of these topologies.

Furthermore, in a GWAS study of the Mexican population, MAC4T was the only gene that was found significantly associated with neurocognitive disorders [57]. CEBPG can form stable heterodimers with CEBPB, a crucial regulator of synaptic plasticity and memory formation. VDAC3 plays a role in synaptic transmission and learning processes.

### 2.14 Genes with human‐specific SVA insertion into its proximal regulatory region

SINE‐R/VNTR/Alu (SVA) is a new and active kind of non‐LTR retrotransposon, mainly found in Hominidae. SVA has about 4933 copies in the human genome, which is about 0.1% of the whole genome, and it is reported that more than 30% of these copies are human‐specific [21].

Similar to the Alu element, in the genes that rank high in the human 4th and 9th levels compared to the three apes, we filtered out those with human‐specific SVA insertions in the proximal regulatory regions. The results are as follows: at the 4th level, we identified the gene RTP3; and at the 9th level, we found ERBB3, HYAL1, RTP3, and RNASE10 (Table 3). RTP3 can promote functional cell surface expression of the bitter taste receptors TAS2R16 and TAS2R43. ERBB3 is involved in several pathways related to the nervous system. Figure S9 shows the UCSC browser snapshot of the human‐specific SVA insertion of ERBB3. HYAL1 is related to myelination [58].

Of note, the motifs present on SVA are quite similar to those present on Alu, as their Jaccard similarity coefficient reaches 0.794. Mutations related to SVA and Alu thus make changes to the CREF matrix jointly.

### 2.15 Human intelligence evolved from saltations

Now we recapitulate what we discovered about human intelligence based on the regulatory sequences in the publicly available genomes of the four hominid species without any a priori. By constructing and comparing their CREF matrices that are further represented by the dual eigen‐modules, we identified two saltations: one between the 4th and 5th and the other between the 9th and 10th levels. The human‐specific cognition and intelligence are thus found from the saltations at the molecular level. The intelligence‐related biological processes enriched at the 4th/9th polarized gene‐eigenvectors are validated by the corresponding *cis*‐regulatory elements on the motif‐eigenvectors. Since the eigen‐modules are orthogonal in the mathematical nature, the two saltations may or may not occur in different time windows in history.

**FIGURE 10:**
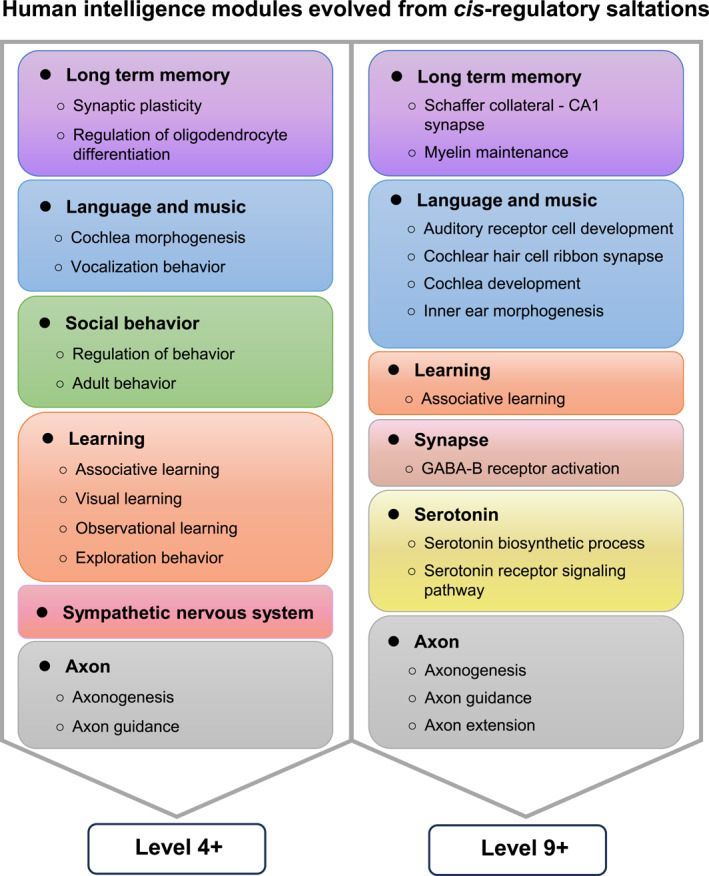
The summarization of human intelligence in the two CREF modules. The 4th module encodes long‐term memory, language and music, social behavior, learning, sympathetic nervous system, and axon; and the 9th module encodes long‐term memory, language and music, learning, synapse, serotonin, and axonogenesis. Of note, only the 4‐th and 9‐th human CREF modules were from saltations, while others are conserved across existing Hominidae species. In other words, the modular intelligence obtained from the human CREF profiles is as shown. The intelligence functions are grouped in different colors. The intelligence phenotypes are defined by the GO, KEGG, and Reactome processes and pathways. The specific genes of these pathways can be found in Tables 1, 2, S2, and S3.

The key features of the two modules are highlighted in Figure 10. Long‐term memory characterized by synaptic plasticity, myelination, and Schaffer collaterals shows up at both levels. Respectively at levels 4 and 9, we found cochlea morphogenesis and inner ear morphogenesis that enable the development of human language and music. Social behavior, significant at level 4, makes it possible for us to live together peacefully and to work collaboratively. Compared to visual and observational learning that show up at level 4, advanced associative learning is found at both levels.

Particularly interesting are the exploratory behavior at level 4, the GABA‐B receptor activation, and serotonin biosynthesis and signaling at level 9. Exploratory behavior is crucial for humans’ creativity. The GABA‐B receptor activation protects our neurons [59]. Serotonin regulates our mood as well as our sleep, appetite, digestion, learning ability, and memory, or in short, our happiness.

## 3 DISCUSSION

### 3.1 Gradualism and its mathematical theories

Although Darwin did not deny the existence of jumps, he held the view that most evolutionary changes proceeded gradually. Darwin did not formulate his discoveries and his views in a mathematical setting as Isaac Newton did for mechanics. Fortunately, his successors such as R. A. Fisher, among others, were good mathematical modelers. The Fisher–Wright model has been the basis of population genetics since its inception. Later, diffusion equation was introduced to calculate the distribution of gene frequencies in populations. Recent important developments in population genetics include coalescent theory [60]. There are other theoretical developments as well [61].

Based on these stochastic models of population genetics, statistical tests were developed to study natural selection. In the 1960s, Kimura and others found that most of the genetic variation, both within and between species, is due to the random drift of alleles that are selectively neutral or nearly neutral. These were established as the neutral theory of molecular evolution [62].

### 3.2 Saltation

In spite of the rich mathematical theories that support gradualism, jumps in the phenotypes of closely related species do exist. Human intelligence is such an instance. Various versions of saltation were proposed in the literature, and most of them were descriptive. The geneticist Richard Goldschmidt coined the term “hopeful monster” to represent macromutations, or large mutations, that lead to speciation. However, not a single mathematical theory of saltation had been found before the CREF regulatory module was proposed. In fact, a mathematical framework able to describe both gradualism and saltation is highly anticipated.

### 3.3 Ising model

A similar puzzle existed in physics about 100 years ago. Professor Curie found that ferromagnetic materials such as iron lose their spontaneous magnetism as the temperature goes beyond a critical point. Mathematical physicists felt urged to find a model that can explain the phase transition. The Ising model came to the need. The research on the Ising model is both tortuous and dramatic. The model was originally invented by the physicist Wilhelm Lenz (1920), whose student Ising (1925) demonstrated in his PhD thesis no phase transition in the 1‐D case. Peierls (1936) offered an argument that phase transition can occur in 2‐D and 3‐D Ising models. Kramers and Wannier (1941) defined a transfer matrix so that the partition function was calculated [63]. Consequently, the critical temperature $T_{c}$ was derived. Later, a series of work by Onsager (1944) [64], Kauffman and Onsager (1948) [65], Yang (1952) [66], etc, showed that phase transition occurs in 2‐D by giving its exact solution. When $T<T_{c}$, the spontaneous magnetization is positive; when $T=T_{c}$, it becomes zero [67, 68].

These mathematical results on the Ising model suggest that the phase transition of a particle system needs certain prerequisites. First, the dimension of the particle interaction is no less than two. Second, the number of particles should be sufficiently large. Third, the strength of interactions between particles alters in a certain way. In the case of the Ising model, it is the temperature that causes the alteration. Mathematically, Kac and Thompson (1966) showed a simple mathematical explanation of phase transition, that is, the asymptotic degeneracy of eigenvalues [69]. In the case of the Ising model, it is the degeneracy of the first and second eigenvalues of the transfer matrix.

### 3.4 Central dogma and protein–DNA interactions

Coming back to the saltation of genomes, we take a view of systems biology according to the insights from the Ising model. First, we include all the annotated genes of a specific genome. The human genome has about 20,000 protein‐coding genes so that the molecular number is sufficiently large. Second, among various kinds of molecular interactions in a cell, those between the *trans*‐factor proteins and DNA *cis*‐elements were the key to mRNA transcription. The central dogma of molecular biology proposed by Francis Crick (1958) states that the information can only be transferred from nucleic acid to nucleic acid [70], or from nucleic acid to protein, but not from protein to protein, or from protein to nucleic acid. In other words, the genetic information can only be multiplied within the domain of nucleic acids. Whereas DNA replication is the way for cell proliferation, transcription of RNA is the only mechanism of information multiplication within a cell.

Regulation of gene transcription is thus crucial for cellular and development biology. The binding of *trans*‐factors to the *cis*‐elements near the TSSs of genes is the central step in regulation. Although the DNA sequence is linear, the 3‐D genomic interactions are ubiquitous, as evidenced by Hi‐C data. On top of that, the *cis*‐*trans* interaction with DNA genes is obviously a 3‐D kind. The strength of *cis*‐*trans* interactions, as we argued earlier in this article, is measured by the number of *cis*‐elements. Putting together, the transcription regulated by DNA–protein interaction in a cell can be represented simply by a CREF matrix, each entry of which corresponds to the occurrences of a *cis*‐element in the proximal regulatory region of a gene.

### 3.5 Switch between quantitative and qualitative change

Although the dual *cis*‐regulatory eigen‐module of a species depicts one static picture of its genome, we can infer the evolutionary dynamics by comparing the CREF modules of several related species in a family. During the majority of history when the relative distance between adjacent eigenvalues was large, the eigen‐structure was fairly stable. Consequently, changes in the CREF matrices only reduced or increased the distances between adjacent eigenvalues and had hardly any influence on the eigenvectors. They belong to the quantitative changes as Hegel coined [71]. As the quantitative changes accumulated, the distance between a pair of adjacent eigenvalues approached zero, and changes in the CREF matrices would lead to large perturbations in the eigenvectors. The changes near the degenerate point became qualitative. This qualitative change is a synonym for phase transition called by physicists. After the new eigenvector is selected and fixed, quantitative changes would play a major role again if the two eigenvalues move apart. Although gradual evolution might be the only mode in the mutations of protein sequences, the evolution of gene regulation has both gradual and saltational modes, which could be explained by the same framework of CREF modules.

### 3.6 CREF matrix versus *cis*‐regulatory element incidence matrix

One motivation for the use of CREF matrices is the ubiquitous multiplicity of *cis*‐elements in the proximal regulatory regions, as demonstrated by Figure 3. On the other hand, it is curious to know what would happen if we replace frequencies by incidences in the regulator‐target matrix. That is, we consider the *cis*‐regulatory element incidence (CREI) matrix *F* = [*f*_*ij*_] in which the entry *f*_*ij*_ is one if a motif *j* is present in the regulatory region of gene *i* and is zero otherwise. In her thesis, Dr. Liang Li reported that the eigen‐decompositions of the Hominidae CREI matrices do not have the conservation and saltations found in those of CREF matrices [24]. The result justifies the CREF regulatory modules to some extent. However, the biological first principle that governs the CREF module is yet to be discovered. The recent single‐cell work [19] strongly supports that the multiplicity of transcription factor binds is crucial for gene regulation.

### 3.7 Human intelligence identified by CREF modules

The measurement of intelligence and cognition differences between humans and apes is a challenging problem. While differences in some aspects such as the brain volume can be measured reliably, other comparisons could be confounded with factors including nutrition and education. Research in paleoanthropology offers some clues to the evolution of human intelligence, but the complete picture is lacking due to the limited evidence. The sequencing of DNA fragments in ancient human remains has become an active research area in recent years. Their implication in inferring human intelligence is challenged by the difficulty in retrieving the transposable repetitive sequences, which were found critical for gene regulation as we reported. Thus, the human cognition identified by the approach of CREF modules, which are obtained from the genomes of existing hominid species, is a fair and reliable discovery at the molecular level.

## 4 CONCLUSION

Not only protein sequences but also gene regulation plays a role in human traits through development. The cognition and intelligence unique to humans can, by and large, be identified using solely the *cis*‐regulatory element frequency profiles without the assistance of protein structures or sequences. From apes to humans, the 4th and 9th eigenvectors underwent saltations. With reference to chimpanzees, the human unique cognition and intelligence include long‐term memory that is characterized by synapsis plasticity and myelination; language and music based on cochlea morphogenesis and vocalization behavior; social behavior that allows us to live together peacefully and to work collaboratively; visual, observational, and associative learning; and sympathetic nervous system. Besides, in the human‐specific CREF modules, we found exploratory behavior crucial for our creativity, the GABA‐B receptor activation that protects our neurons, and serotonin biosynthesis/signaling that regulates our happiness. The transitions in the CREF modules were driven by Alu and SVA direct insertions along with other factors. Since the CREF modules are based on the genomes of existing species, the inference is reliable. Although gradual evolution might be the only mode in the mutations of protein sequences, the evolution of gene regulation has both gradual and saltational modes, which could be explained by the framework of CREF eigen‐modules.

## AUTHOR CONTRIBUTIONS

**Xiaojie Li**: Data curation; Formal analysis; Investigation; Software; Validation; Visualization; Writing ‐ original draft; Writing ‐ review & editing. **Jianhui Shi**: Data curation; Formal analysis; Investigation; Software; Validation; Visualization; Writing ‐ original draft; Writing ‐ review & editing. **Lei M. Li**: Conceptualization; Formal analysis; Funding acquisition; Investigation; Methodology; Project administration; Resources; Supervision; Validation; Visualization; Writing ‐ original draft; Writing ‐ review & editing.

## CONFLICT OF INTEREST STATEMENT

The authors Xiaojie Li, Jianhui Shi, and Lei M. Li declare that they have no conflicts of interest.

## ETHICS STATEMENT

This article does not contain any studies with human or animal subjects performed by any of the authors.

## Supporting information

Supplementary material

## Data Availability

All data analyzed during this study are included in this article and its supplementary materials file. The supplementary materials file can be found online with this article.

## References

[1] King MC , Wilson AC . Evolution at two levels in humans and chimpanzees. Science. 1975;188(4184):107–116. 1090005 10.1126/science.1090005

[2] Cao J , Luo Z , Cheng Q , Xu Q , Zhang Y , Wang F , et al. Three‐dimensional regulation of transcription. Protein Cell. 2015;6(4):241–253. 25670626 10.1007/s13238-015-0135-7 PMC4383755

[3] Lee TI , Young RA . Transcriptional regulation and its misregulation in disease. Cell. 2013;152(6):1237–1251. 23498934 10.1016/j.cell.2013.02.014 PMC3640494

[4] Mitsis T , Efthimiadou A , Bacopoulou F , Vlachakis D , Chrousos GP , Eliopoulos E . Transcription factors and evolution: an integral part of gene expression (Review). World Academy of Sciences Journal. 2020;2(1):3–8.

[5] Wray GA . The evolutionary significance of *cis*‐regulatory mutations. Nat Rev Genet. 2007;8(3):206–216. 17304246 10.1038/nrg2063

[6] Ewens WJ , Grant GR . Statistical methods in bioinformatics: an introduction. 2nd ed. New York: Springer; 2005. p. 597. (Statistics for biology and health).

[7] Ewens WJ . Mathematical population genetics. 1: theoretical introduction. 2. ed. New York: Springer; 2004. p. 417. (Interdisciplinary applied mathematics Mathematical biology).

[8] Horton CA , Alexandari AM , Hayes MGB , Marklund E , Schaepe JM , Aditham AK , et al. Short tandem repeats bind transcription factors to tune eukaryotic gene expression. Science. 2023;381(6664):eadd1250. 37733848 10.1126/science.add1250

[9] Wright SE , Todd PK . Native functions of short tandem repeats. Elife. 2023;12:e84043. 36940239 10.7554/eLife.84043 PMC10027321

[10] Polak P , Domany E . Alu elements contain many binding sites for transcription factors and may play a role in regulation of developmental processes. BMC Genom. 2006;7:133. 10.1186/1471-2164-7-133 PMC1513395 16740159

[11] Li L , Zhang S , Li LM . Dual eigen‐modules of *cis* ‐element regulation profiles and selection of cognition‐language eigen‐direction along evolution in Hominidae. Mol Biol Evol. 2020;37(6):1679–1693. 32068872 10.1093/molbev/msaa036 PMC10615152

[12] Kumar S , Stecher G , Suleski M , Hedges SB . TimeTree: a resource for timelines, timetrees, and divergence times. Mol Biol Evol. 2017;34(7):1812–1819. 28387841 10.1093/molbev/msx116

[13] Doniger SW , Fay JC . Frequent gain and loss of functional transcription factor binding sites. PLoS Comput Biol. 2007;3(5):e99. 17530920 10.1371/journal.pcbi.0030099 PMC1876492

[14] Matys V . TRANSFAC(R) and its module TRANSCompel(R): transcriptional gene regulation in eukaryotes. Nucleic Acids Res. 2006;34(90001):D108–D110. 16381825 10.1093/nar/gkj143 PMC1347505

[15] Kel AE , Gößling E , Reuter I , Cheremushkin E , Kel‐Margoulis OV , Wingender E . MATCHTM: a tool for searching transcription factor binding sites in DNA sequences. Nucleic Acids Res. 2003;31(13):3576–3579. 12824369 10.1093/nar/gkg585 PMC169193

[16] Zhang C , Xuan Z , Otto S , Hover JR , McCorkle SR , Mandel G , et al. A clustering property of highly‐degenerate transcription factor binding sites in the mammalian genome. Nucleic Acids Res. 2006;34(8):2238–2246. 16670430 10.1093/nar/gkl248 PMC1456330

[17] Paixão T , Azevedo RBR . Redundancy and the evolution of *cis*‐regulatory element multiplicity. PLoS Comput Biol. 2010;6(7):e1000848. 20628617 10.1371/journal.pcbi.1000848 PMC2900288

[18] Xu H , Sepúlveda LA , Figard L , Sokac AM , Golding I . Combining protein and mRNA quantification to decipher transcriptional regulation. Nat Methods. 2015;12(8):739–742. 26098021 10.1038/nmeth.3446 PMC4521975

[19] Doughty BR , Hinks MM , Schaepe JM , Marinov GK , Thurm AR , Rios‐Martinez C , et al. Single‐molecule chromatin configurations link transcription factor binding to expression in human cells. 2024. Preprint at bioRxiv:2024.02.02.578660.

[20] Feng Y , Zhang S , Li L , Li LM . The *cis*‐trans binding strength defined by motif frequencies facilitates statistical inference of transcriptional regulation. BMC Bioinf. 2019;20(7):201. 10.1186/s12859-019-2732-6 PMC6509875 31074378

[21] Tang W , Mun S , Joshi A , Han K , Liang P . Mobile elements contribute to the uniqueness of human genome with 15,000 human‐specific insertions and 14 Mbp sequence increase. DNA Res. 2018;25(5):521–533. 30052927 10.1093/dnares/dsy022 PMC6191304

[22] Lin Z , Chen M , Ma Y . The augmented Lagrange multiplier method for exact recovery of corrupted low‐rank matrices. J Struct Biol. 2013;181(2):116–127.23110852

[23] Stewart GW , Sun Jguang . Matrix perturbation theory. Boston: Academic Press; 1990. p. 365. (Computer science and scientific computing).

[24] Li L . The evolution of transcription regulation in Hominidae from the perspective of *cis*‐element frequency [Ph.D. Thesis]. University of Chinese Academy of Sciences; 2021.

[25] Eigenvalue perturbation. In: Wikipedia [Internet]. 2024 [cited 2024 Aug 22].

[26] Cheng C , Fabrizio P , Ge H , Wei M , Longo VD , Li LM . Significant and systematic expression differentiation in long‐lived yeast strains. PLoS One. 2007;2(10):e1095. 17971858 10.1371/journal.pone.0001095 PMC2039703

[27] Morris WE , Brown CR . David Hume. In: Zalta EN , Nodelman U , editors. The stanford encyclopedia of philosophy [Internet]. Winter 2023. Metaphysics Research Lab, Stanford University; 2023. [cited 2024 Jun 29].

[28] Gruber MJ , Ranganath C . How curiosity enhances hippocampus‐dependent memory: the prediction, appraisal, curiosity, and exploration (PACE) framework. Trends Cognit Sci. 2019;23(12):1014–1025. 31706791 10.1016/j.tics.2019.10.003 PMC6891259

[29] Serotonin. In: Wikipedia [internet]. 2024 [cited 2024 Jun 25].

[30] Flitton M , Rielly N , Warman R , Warden D , Smith AD , Macdonald IA , et al. Interaction of nutrition and genetics via DNMT3L‐mediated DNA methylation determines cognitive decline. Neurobiol Aging. 2019;78:64–73. 30877840 10.1016/j.neurobiolaging.2019.02.001

[31] Zhang B , Wang Q , Miao T , Yu B , Yuan P , Kong J , et al. Whether Alzheimer’s diseases related genes also differently express in the hippocampus of Ts65Dn mice? Int J Clin Exp Pathol. 2015;8(4):4120–4125. 26097601 PMC4466988

[32] Collins SM , Belagodu AP , Reed SL , Galvez R . SHANK1 is differentially expressed during development in CA1 hippocampal neurons and astrocytes. Dev Neurobiol. 2018;78(4):363–373. 29218848 10.1002/dneu.22564

[33] Södersten E , Lilja T , Hermanson O . The novel BTB/POZ and zinc finger factor Zbtb45 is essential for proper glial differentiation of neural and oligodendrocyte progenitor cells. Cell Cycle. 2010;9(24):4866–4875. 21131782 10.4161/cc.9.24.14154 PMC3047810

[34] Plitt MH , Kaganovsky K , Südhof TC , Giocomo LM . Hippocampal place code plasticity in CA1 requires postsynaptic membrane fusion. 2023. Preprint at bioRxiv:2023.11.20.567978. 10.1016/j.neuron.2026.03.006 PMC13488814 41932328

[35] Roy ER , Chiu G , Li S , Propson NE , Kanchi R , Wang B , et al. Concerted type I interferon signaling in microglia and neural cells promotes memory impairment associated with amyloid β plaques. Immunity. 2022;55(5):879–894.e6. 35443157 10.1016/j.immuni.2022.03.018 PMC9109419

[36] Ma Z , Zeng Y , Wang M , Liu W , Zhou J , Wu C , et al. N4BP1 mediates RAM domain‐dependent notch signaling turnover during neocortical development. EMBO J. 2023;42(22):e113383. 37807845 10.15252/embj.2022113383 PMC10646556

[37] Woodward KJ , Stampalia J , Vanyai H , Rijhumal H , Potts K , Taylor F , et al. Atypical nested 22q11.2 duplications between LCR22B and LCR22D are associated with neurodevelopmental phenotypes including autism spectrum disorder with incomplete penetrance. Mol Genet Genomic Med. 2019;7(2):e00507. 30614210 10.1002/mgg3.507 PMC6393688

[38] Kong X , Shu X , Wang J , Liu D , Ni Y , Zhao W , et al. Fine‐tuning of mTOR signaling by the UBE4B‐KLHL22 E3 ubiquitin ligase cascade in brain development. Development. 2022;149(24):dev201286. 36440598 10.1242/dev.201286 PMC9845739

[39] Neyazi B , Tanrikulu L , Wilkens L , Hartmann C , Stein KP , Dumitru CA , et al. Procollagen‐lysine, 2‐oxoglutarate 5‐dioxygenase 2 expression in brain arteriovenous malformations and its association with brain arteriovenous malformation size. World Neurosurg. 2017;102:79–84. 28279775 10.1016/j.wneu.2017.02.116

[40] Li M , Yue W . VRK2, a candidate gene for psychiatric and neurological disorders. Mol Neuropsychiatry. 2018;4(3):119–133. 30643786 10.1159/000493941 PMC6323383

[41] Yoshihara T , Satake H , Nishie T , Okino N , Hatta T , Otani H , et al. Lactosylceramide synthases encoded by B4galt5 and 6 genes are pivotal for neuronal generation and myelin formation in mice. PLoS Genet. 2018;14(8):e1007545. 30114188 10.1371/journal.pgen.1007545 PMC6095488

[42] Alberini CM . Transcription factors in long‐term memory and synaptic plasticity. Physiol Rev. 2009;89(1):121–145. 19126756 10.1152/physrev.00017.2008 PMC3883056

[43] Okamoto Sichi , Sherman K , Bai G , Lipton SA . Effect of the ubiquitous transcription factors, SP1 and MAZ, on NMDA receptor subunit type 1 (NR1) expression during neuronal differentiation. Brain Res Mol Brain Res. 2002;107(2):89–96. 12425938 10.1016/s0169-328x(02)00440-0

[44] Alsiö JM , Tarchini B , Cayouette M , Livesey FJ . Ikaros promotes early‐born neuronal fates in the cerebral cortex. Proc Natl Acad Sci USA. 2013;110(8):E716. 23382203 10.1073/pnas.1215707110 PMC3581915

[45] Agoston DV , Szemes M , Dobi A , Palkovits M , Georgopoulos K , Gyorgy A , et al. Ikaros is expressed in developing striatal neurons and involved in enkephalinergic differentiation. J Neurochem. 2007;102(6):1805–1816. 17504264 10.1111/j.1471-4159.2007.04653.x

[46] Sakai H , Fujii Y , Kuwayama N , Kawaji K , Gotoh Y , Kishi Y . Plag1 regulates neuronal gene expression and neuronal differentiation of neocortical neural progenitor cells. Gene Cell. 2019;24(10):650–666. 10.1111/gtc.12718 31442350

[47] Qin S , Zhang CL . Role of Kruppel‐like factor 4 in neurogenesis and radial neuronal migration in the developing cerebral cortex. Mol Cell Biol. 2012;32(21):4297–4305. 22907754 10.1128/MCB.00838-12 PMC3486145

[48] Qin S , Liu M , Niu W , Zhang CL . Dysregulation of Kruppel‐like factor 4 during brain development leads to hydrocephalus in mice. Proc Natl Acad Sci USA. 2011;108(52):21117–21121. 22160720 10.1073/pnas.1112351109 PMC3248552

[49] Topilko P , Schneider‐Maunoury S , Levi G , Baron‐Van Evercooren A , Chennoufi AB , Seitanidou T , et al. Krox‐20 controls myelination in the peripheral nervous system. Nature. 1994;371(6500):796–799. 7935840 10.1038/371796a0

[50] Cubelos B , Sebastián‐Serrano A , Beccari L , Calcagnotto ME , Cisneros E , Kim S , et al. Cux1 and Cux2 regulate dendritic branching, spine morphology, and synapses of the upper layer neurons of the cortex. Neuron. 2010;66(4):523–535. 20510857 10.1016/j.neuron.2010.04.038 PMC2894581

[51] Yu WM , Appler JM , Kim YH , Nishitani AM , Holt JR , Goodrich LV . A Gata3‐Mafb transcriptional network directs post‐synaptic differentiation in synapses specialized for hearing. Elife. 2013;2:e01341. 24327562 10.7554/eLife.01341 PMC3851837

[52] Zhang T , Xu J , Maire P , Xu PX . Six1 is essential for differentiation and patterning of the mammalian auditory sensory epithelium. PLoS Genet. 2017;13(9):e1006967. 28892484 10.1371/journal.pgen.1006967 PMC5593176

[53] Zuber ME , Gestri G , Viczian AS , Barsacchi G , Harris WA . Specification of the vertebrate eye by a network of eye field transcription factors. Development. 2003;130(21):5155–5167. 12944429 10.1242/dev.00723

[54] Li LM , Li M , Li L . *Cis*‐regulatory element frequency modules and their phase transition across Hominidae. In: Lu HHS , Schölkopf B , Wells MT , Zhao H , editors. Handbook of statistical bioinformatics [Internet]. Berlin: Springer; 2022. p. 371–395. [cited 2024 Jun 18].

[55] Harris RA , Raveendran M , Worley KC , Rogers J . Unusual sequence characteristics of human chromosome 19 are conserved across 11 nonhuman primates. BMC Evol Biol. 2020;20(1):33. 32106815 10.1186/s12862-020-1595-9 PMC7045612

[56] Peng Y , Bjelde A , Aceituno PV , Mittermaier FX , Planert H , Grosser S , et al. Directed and acyclic synaptic connectivity in the human layer 2‐3 cortical microcircuit. Science. 2024;384(6693):338–343. 38635709 10.1126/science.adg8828

[57] Bliskunova T , Genis‐Mendoza AD , Martínez‐Magaña JJ , Vega‐Sevey JG , Jiménez‐Genchi J , Roche A , et al. Association of MGAT4C with major neurocognitive disorder in the Mexican population. Gene. 2021;778:145484. 33581268 10.1016/j.gene.2021.145484

[58] Preston M , Gong X , Su W , Matsumoto SG , Banine F , Winkler C , et al. Digestion products of the PH20 hyaluronidase inhibit remyelination. Ann Neurol. 2013;73(2):266–280. 23463525 10.1002/ana.23788 PMC3608752

[59] Tu H , Xu C , Zhang W , Liu Q , Rondard P , Pin JP , et al. GABAB receptor activation protects neurons from apoptosis via IGF‐1 receptor transactivation. J Neurosci. 2010;30(2):749–759. 20071540 10.1523/JNEUROSCI.2343-09.2010 PMC6633015

[60] Kingman JFC . Origins of the coalescent 1974–1982. Genetics. 2000;156:1461–1463. 11102348 10.1093/genetics/156.4.1461 PMC1461350

[61] Ao P . Laws in Darwinian evolutionary theory. Phys Life Rev. 2005;2(2):117–156.

[62] Kimura M . The neutral theory of molecular evolution. Cambridge: Cambridge University Press; 1983.

[63] Kramers HA , Wannier GH . Statistics of the two‐dimensional ferromagnet. Part II. Phys Rev. 1941;60(3):263–276.

[64] Onsager L . Crystal statistics. I. A two‐dimensional model with an order‐disorder transition. Phys Rev. 1944;65(3–4):117–149.

[65] Kaufman B , Onsager L . Crystal statistics. III. Short‐range order in a binary ising lattice. Phys Rev. 1949;76(8):1244–1252.

[66] Yang CN . The spontaneous magnetization of a two‐dimensional ising model. Phys Rev. 1952;85(5):808–816.

[67] Montroll EW , Potts RB , Ward JC . Correlations and spontaneous magnetization of the two‐dimensional ising model. J Math Phys. 1963;4(2):308–322.

[68] Deift P , Its A , Krasovsky I . Toeplitz matrices and toeplitz determinants under the impetus of the ising model: some history and some recent results. Commun Pure Appl Math. 2013;66(9):1360–1438.

[69] Kac M , Thompson CJ . On the mathematical mechanism of phase transition. Proc Natl Acad Sci USA. 1966;55(4):676–683. 16591343 10.1073/pnas.55.4.676 PMC224209

[70] Crick FH . On protein synthesis. Symp Soc Exp Biol. 1958;12:138–163. 13580867

[71] Hegel GWF . In: Di Giovanni G , editor. Georg Wilhelm friedrich Hegel: the science of logic [Internet]. Cambridge: Cambridge University Press; 2010. [cited 2024 Jun 18]. (Cambridge Hegel Translations).

[72] Cheng C , Fabrizio P , Ge H , Wei M , Longo VD , Li LM . Significant and systematic expression differentiation in long‐lived yeast strains. PLoS One. 2007;2(10):e1095. 17971858 10.1371/journal.pone.0001095 PMC2039703

[73] Ashburner M , Ball CA , Blake JA , Botstein D , Butler H , Cherry JM , et al. Gene Ontology: tool for the unification of biology. Nat Genet. 2000;25(1):25–29. 10802651 10.1038/75556 PMC3037419

[74] Aleksander SA , Aleksander SA , Balhoff J , Carbon S , Cherry JM , Drabkin HJ , et al. The gene Ontology knowledgebase in 2023. Genetics. 2023;224(1):iyad031. 36866529 10.1093/genetics/iyad031 PMC10158837

[75] Kanehisa M , Furumichi M , Tanabe M , Sato Y , Morishima K . KEGG: new perspectives on genomes, pathways, diseases and drugs. Nucleic Acids Res. 2017;45(D1):D353–D361. 27899662 10.1093/nar/gkw1092 PMC5210567

[76] Kanehisa M , Furumichi M , Sato Y , Kawashima M , Ishiguro‐Watanabe M . KEGG for taxonomy‐based analysis of pathways and genomes. Nucleic Acids Res. 2023;51(D1):D587–D592. 36300620 10.1093/nar/gkac963 PMC9825424

[77] Milacic M , Beavers D , Conley P , Gong C , Gillespie M , Griss J , et al. The reactome pathway knowledgebase 2024. Nucleic Acids Res. 2024;52(D1):D672–D678. 37941124 10.1093/nar/gkad1025 PMC10767911

[78] Bao W , Kojima KK , Kohany O . Repbase Update, a database of repetitive elements in eukaryotic genomes. Mobile DNA. 2015;6(1):11. 26045719 10.1186/s13100-015-0041-9 PMC4455052

