# Supplementary material for: The human intelligence evolved from proximal cis‐regulatory saltations
Source: Quant Biol. 2025 Jan 3;13(2):e88. doi: 10.1002/qub2.88 (PMC12806144; doi:10.1002/qub2.88)
Supplement: Supplementary file 1 — Supplementary material [file QUB2-13-e88-s001.pdf]

The Human Intelligence Evolved from Proximal Cis-regulatory Saltations

Xiaojie Li[1,2¶], Jianhui Shi[1,2¶], Lei M. Li[1,2*]

[1] Academy of Mathematics and Systems Science, Chinese Academy of Sciences, Beijing, China

[2] School of Mathematical Sciences, University of the Chinese Academy of Sciences, Beijing, China

*** Corresponding author, E-mail: lilei@amss.ac.cn**

[¶]These authors contributed equally to this work.

Contents

Fig. S1. 2

Fig. S2. 3

Fig. S3. 4

Fig. S4. 5

Fig. S5. 6

Fig. S6. 7

Fig. S7. 8

Fig. S8. 9

Fig. S9. 10

Table S1. 11

Table S1 (continued). 12

Table S2. 13

Table S2 (continued). 14

Table S2 (continued). 15

Table S3. 16

Table S3 (continued). 17

Table S3 (continued). 18

Table S3 (continued). 19

Table S4. 20

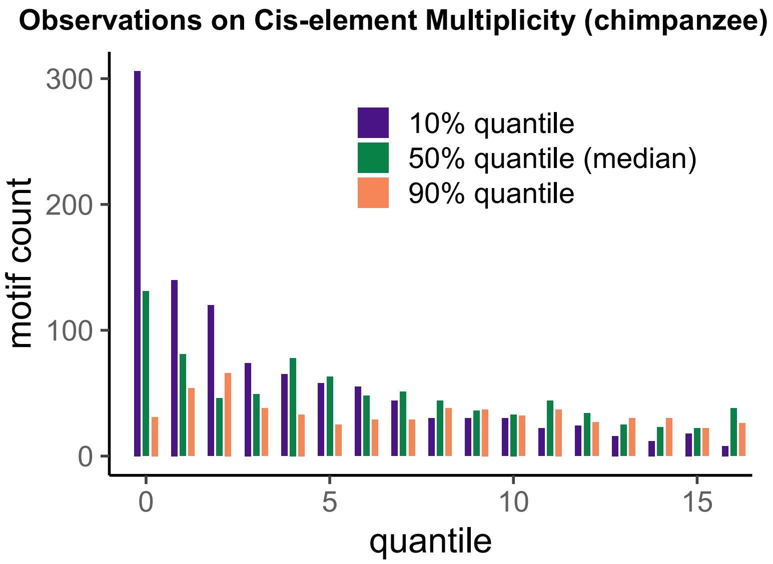

Fig. S1.

The distributions of rounded-down quantiles of 1403 motifs across chimpanzee protein genes ranging from 0 to 20. It is noted that the pattern of cis-element distributions is fairly conserved between human and chimpanzee.

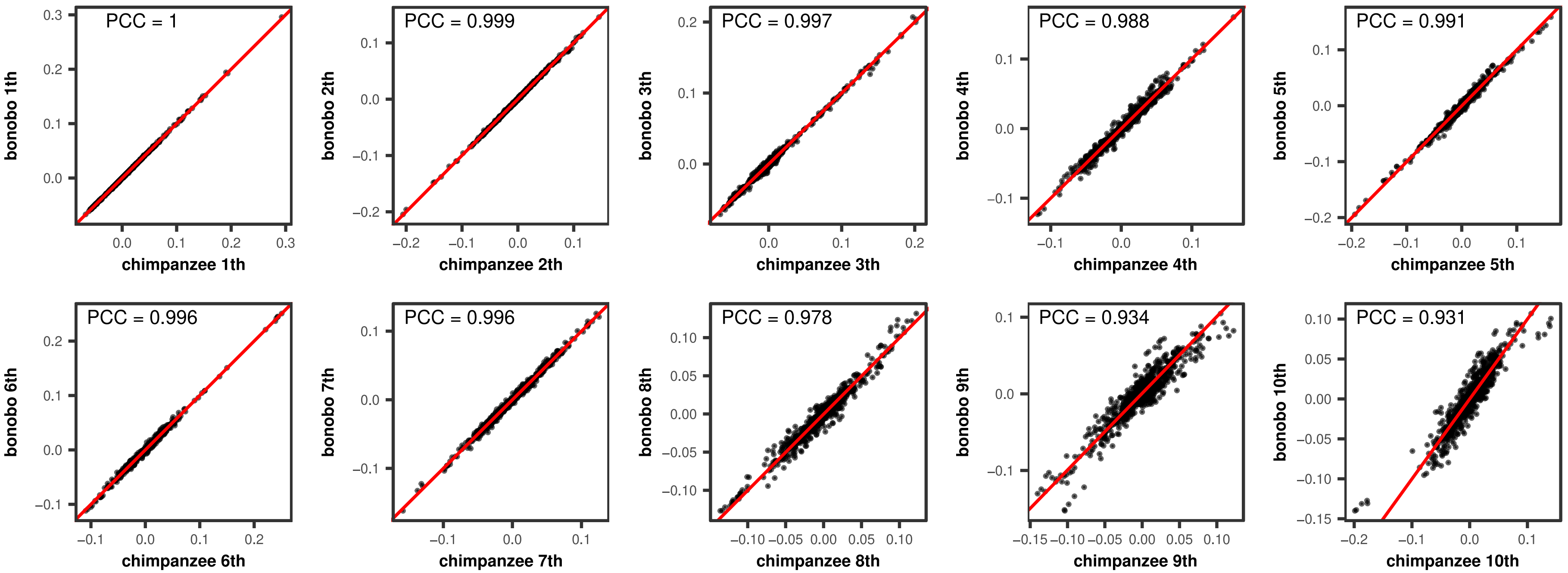

Fig. S2.

The scatter plots of bonobos' motif-eigenvector loadings versus chimpanzees' with fitted Deming regression lines.

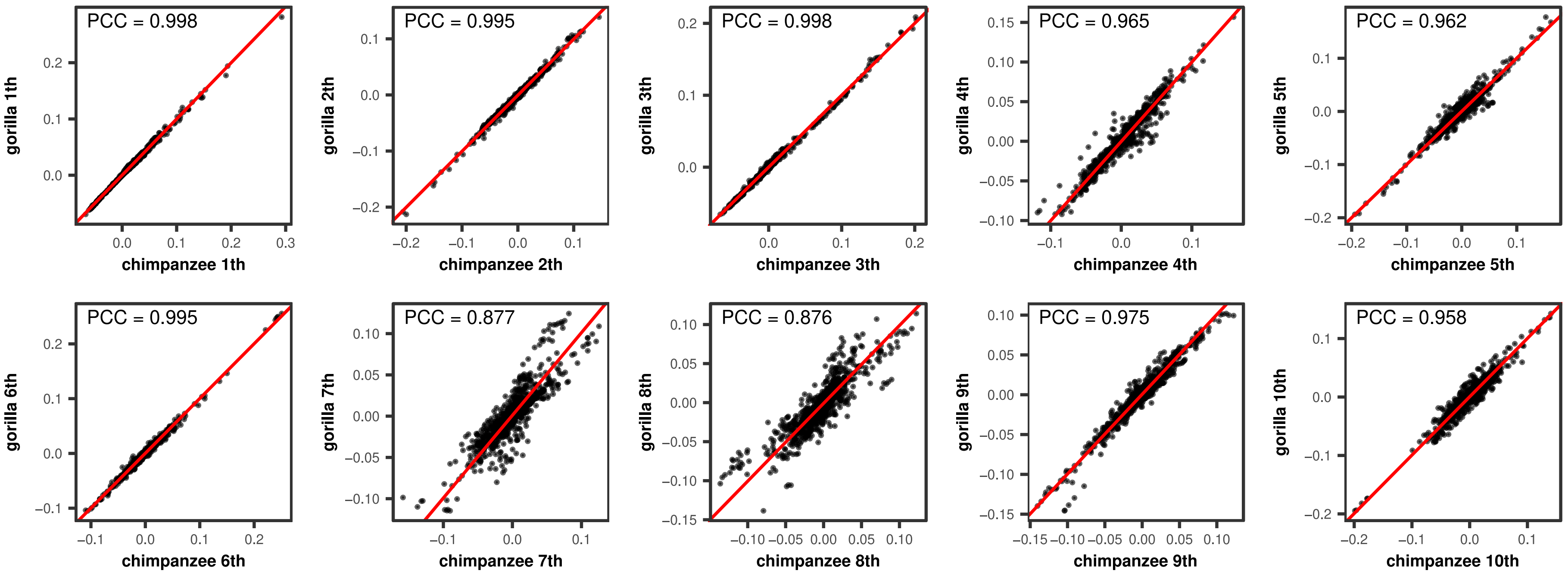

Fig. S3.

The scatter plots of gorillas' motif-eigenvector loadings versus chimpanzees' with fitted Deming regression lines.

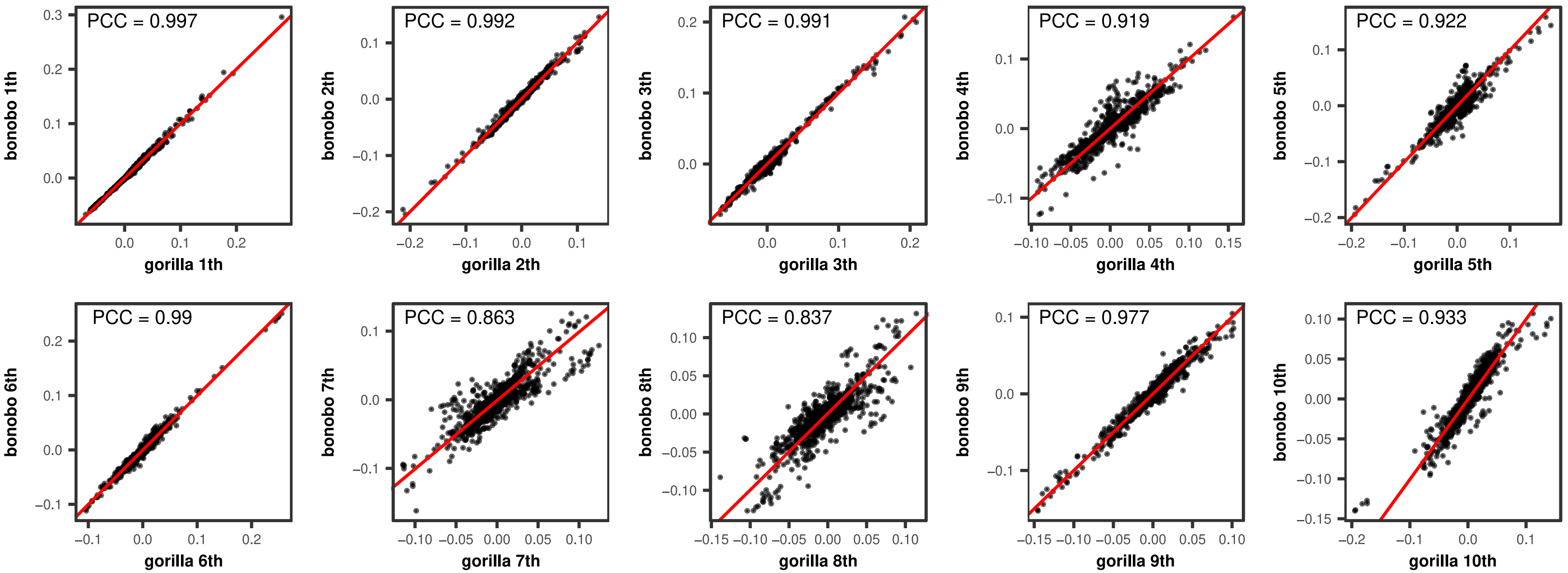

Fig. S4.

The scatter plots of gorillas' motif-eigenvector loadings versus bonobos' with fitted Deming regression lines.

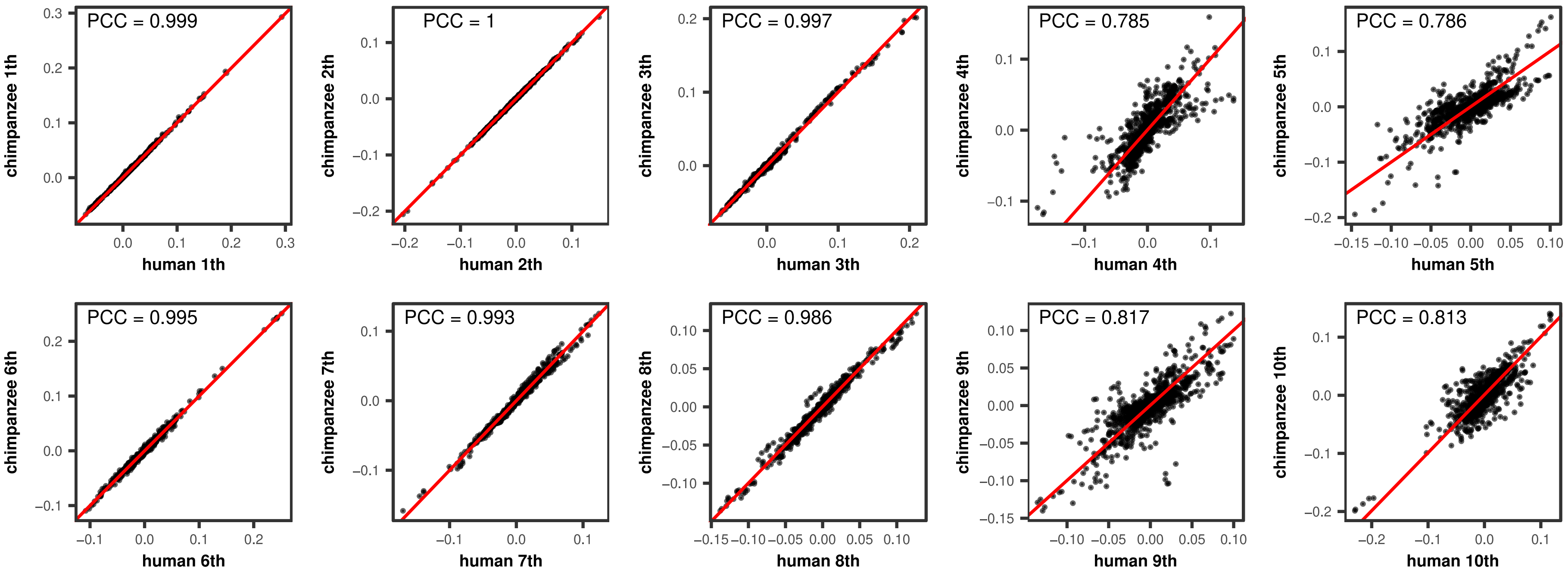

Fig. S5.

The scatter plots of humans' motif-eigenvector loadings versus chimpanzees' with fitted Deming regression lines.

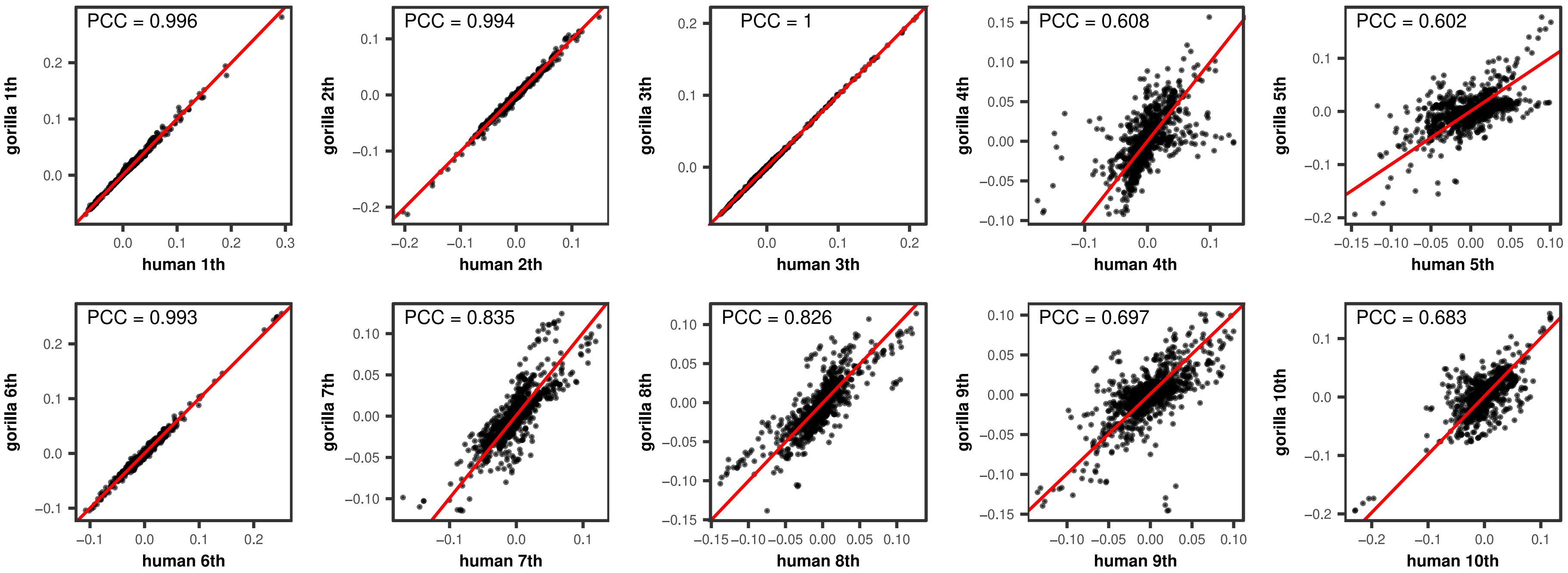

Fig. S6.

The scatter plots of humans' motif-eigenvector loadings versus gorillas' with fitted Deming regression lines.

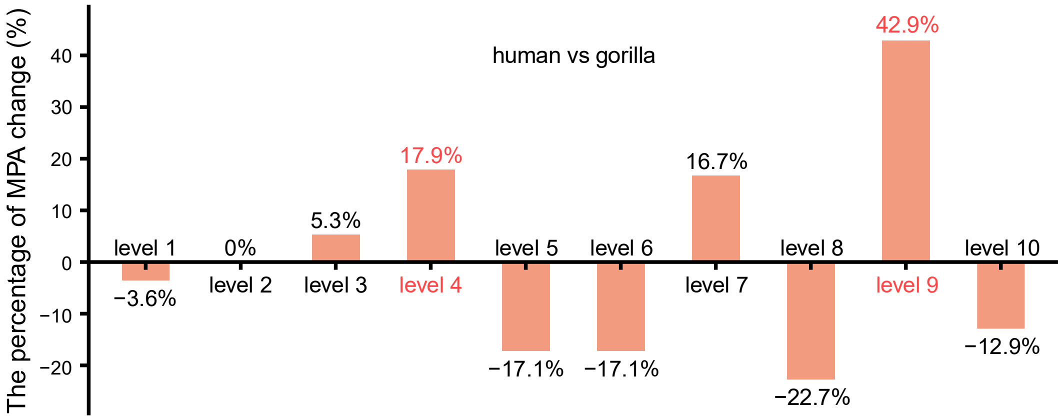

Fig. S7.

The relative change of MPAs (motifs present on Alu elements) in percentages at each level from gorilla to human. The number of MPAs increases most significantly at level 9 by 42.9 %, followed by 17.9 % at level 4.

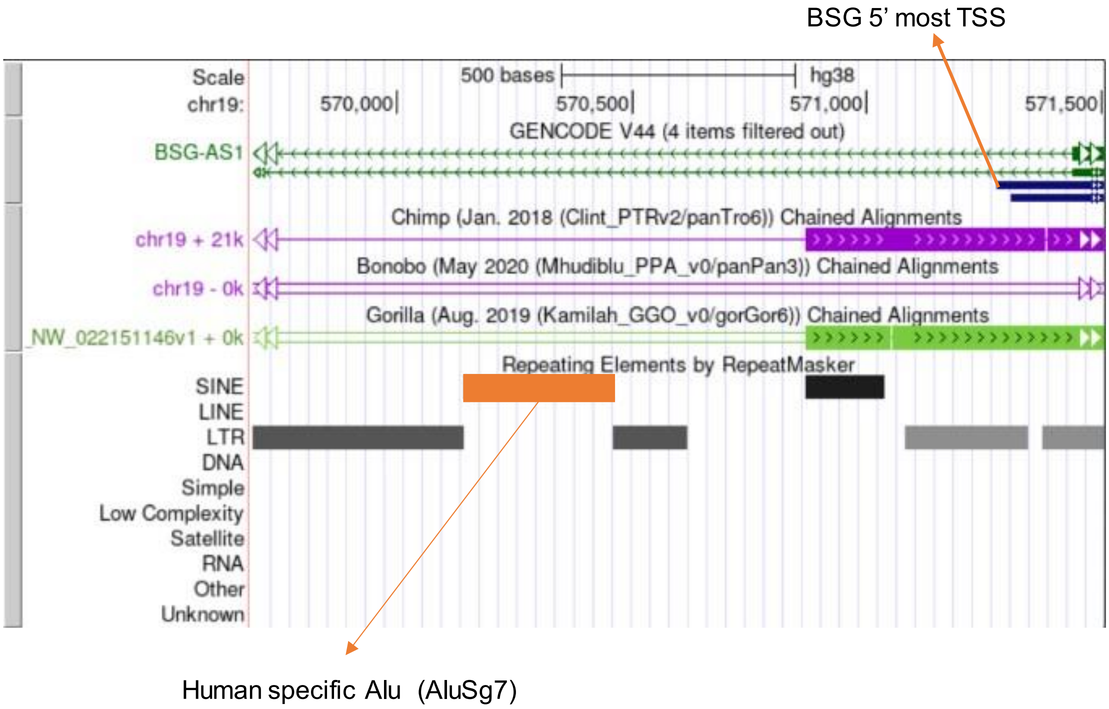

Fig. S8.

The genome alignment of the BSG gene between humans and three apes shown in the UCSC browser. The 5' most TSS position of the human BSG gene and inserted Alu element are marked.

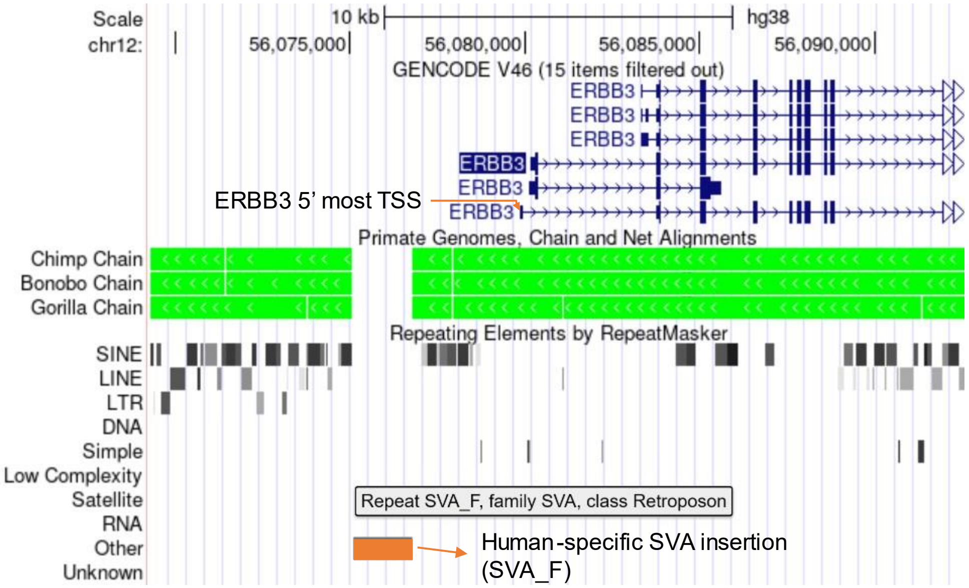

Fig. S9.

The genome alignment of the ERBB3 gene between humans and three apes shown in the UCSC browser. The 5' most TSS position of the human ERBB3 gene and inserted SVA element are marked.

Table S1.

Intelligence and cognition processes enriched at the 4th and 9th CREF eigen-modules.

✧ **Long-term memory**

ID	Description	P-value			
		Human 9+	Chimpanzee 9+	Human 4+	Chimpanzee 4+
GO:0007616	long-term memory	0.282394	0.637449	0.022269	0.119324
GO:0043217	myelin maintenance	0.032153	0.131058	0.258905	0.248669
GO:0048167	regulation of synaptic plasticity	0.024117	0.203978	1.35E-05	0.556382
GO:0098685	Schaffer collateral - CA1 synapse	1.7E-06	0.021843	0.477433	0.455303

✧ **Learning**

GO:0008306	associative learning	0.010789	0.539291	0.043197	0.775601
GO:0008542	visual learning	0.482009	0.978004	0.000174	0.267893
GO:0035640	exploration behavior	0.165599	0.353184	0.002124	0.141408
GO:0042297	vocal learning	0.589264	0.392715	0.076338	0.078603
GO:0098597	observational learning	0.492364	0.64794	0.049542	0.085774

✧ **Social behavior**

GO:0035176	social behavior	0.202997	0.373939	0.000788	0.481361
GO:0030534	adult behavior	0.485539	0.635682	0.034788	0.541787
GO:0050795	regulation of behavior	0.342048	0.300786	0.005328	0.259708

✧ **GABA**

977443	GABA receptor activation	0.003115	0.390347	0.018916	0.203426
977444	GABA B receptor activation	0.012369	0.180675	0.19651	0.854501
991365	Activation of GABAB receptors	0.012369	0.180675	0.19651	0.854501
GO:0004890	GABA-A receptor activity	0.043031	0.893278	0.001858	0.001167
GO:0098982	GABA-ergic synapse	0.000885	0.766029	0.000231	0.012051
GO:1902711	GABA-A receptor complex	0.043049	0.891453	0.001875	0.001141
GO:0051932	synaptic transmission, GABAergic	0.011951	0.943019	0.001348	0.005313
hsa04727	GABAergic synapse	0.016142	0.393574	0.010181	0.023509
GO:0097151	positive regulation of inhibitory postsynaptic potential	0.002723	0.095767	0.028823	0.255769

Table S1 (continued).

Intelligence and cognition processes enriched at the 4th and 9th CREF eigen-modules.

✧ **Serotonin**

181429	Serotonin Neurotransmitter Release Cycle	0.147981	0.63505	0.022781	0.70032
209931	Serotonin and melatonin biosynthesis	0.004625	0.090544	0.857935	0.97211
GO:0007210	serotonin receptor signaling pathway	0.029449	0.105362	0.182834	0.605445
GO:0014062	regulation of serotonin secretion	0.596428	0.380236	0.047389	0.073208
GO:0022850	serotonin-gated cation-selective channel activity	0.033132	0.17475	0.414188	0.832667
GO:0031826	type 2A serotonin receptor binding	0.048347	0.078627	0.066851	0.413093
GO:0042427	serotonin biosynthetic process	0.006024	0.170031	0.257555	0.713657
GO:1904602	serotonin-activated cation-selective channel complex	0.032903	0.17209	0.41494	0.830521

✧ **Axon**

GO:0007409	axonogenesis	0.000135	0.320911	0.007025	0.024507
GO:0007411	axon guidance	0.000434	0.31861	5.23E-05	0.079845
GO:0030424	axon	0.000116	0.019711	0.007669	0.028756
GO:0048675	axon extension	0.017767	0.386717	0.069245	0.339462
GO:0048680	positive regulation of axon regeneration	0.031574	0.155013	0.080382	0.15873
GO:0050770	regulation of axonogenesis	5.4E-05	0.035462	0.138024	0.57971
GO:1902379	chemoattractant activity involved in axon guidance	0.045379	0.577736	0.462204	0.479339

✧ **Sympathetic nervous system**

GO:0048485	sympathetic nervous system development	0.36562	0.384397	0.004612	0.057006

Table S2.
Genes in the enriched gene sets relating to intelligence, ranking in the top 1500 of the human polarized gene-eigenvector at level 4.

Gene Name	Rank	Biological Processes	Process Number
JPH4	12	regulation of synaptic plasticity	1
SYNGAP1	16	visual learning	1
SLC17A7	41	excitatory synapse	1
NFATC4	50	long-term memory	1
MBD5	56	regulation of behavior	1
CAMK2N1	69	long-term memory	1
GRP	78	social behavior	1
SHANK1	91	long-term memory; associative learning; adult behavior; social behavior; vocalization behavior; excitatory synapse	6
SHANK3	100	adult behavior; social behavior; vocalization behavior	3
ADCY6	129	GABA receptor activation	1
NEUROD2	166	associative learning	1
NTF4	177	long-term memory	1
ARHGEF25	184	axon guidance	1
SLC6A1	195	associative learning	1
GRIA3	209	postsynaptic density membrane	1
ADGRB1	215	axonogenesis; regulation of synaptic plasticity	2
ASCL1	218	sympathetic nervous system development	1
HPN	250	cochlea morphogenesis	1
AGER	258	regulation of synaptic plasticity	1
ATXN1	277	social behavior	1
ATP2B4	285	glutamatergic synapse	1
EFNB3	303	axon guidance; postsynaptic density membrane	2
NRXN2	309	adult behavior; social behavior; vocalization behavior	3
GRIN2B	324	regulation of synaptic plasticity; postsynaptic density membrane	2
CACNG8	328	postsynaptic density membrane	1
HTR1A	340	exploration behavior; regulation of behavior	2
NLGN2	345	social behavior; positive regulation of inhibitory postsynaptic potential; presynapse assembly; excitatory synapse	4
RARA	409	regulation of synaptic plasticity	1
SIPA1L1	429	regulation of synaptic plasticity	1

Table S2 (continued).

Genes in the enriched gene sets relating to intelligence, ranking in the top 1500 of the human polarized gene-eigenvector at level 4.

Gene Name	Rank	Biological Processes	Process Number
ATP1A2	450	visual learning	1
NF1	452	visual learning; sympathetic nervous system development; observational learning	3
FZD2	520	cochlea morphogenesis	1
NUMBL	549	axonogenesis	1
SCT	550	regulation of synaptic plasticity	1
CLN3	585	associative learning	1
PCDH17	591	adult behavior	1
RIMS3	597	regulation of synaptic plasticity	1
NTNG1	611	axonogenesis	1
ABAT	628	exploration behavior; positive regulation of inhibitory postsynaptic potential	2
BOC	651	axon guidance	1
BCAN	658	glutamatergic synapse	1
CACNG2	664	postsynaptic density membrane	1
ELFN1	679	excitatory synapse	1
ADCY4	746	GABA receptor activation	1
GNGT2	783	GABA receptor activation	1
MAP1S	803	axonogenesis	1
ARFGEF2	856	glutamatergic synapse	1
GNG12	858	GABA receptor activation	1
ACTN1	901	glutamatergic synapse	1
NRXN1	924	adult behavior; social behavior; vocalization behavior	3
ENAH	952	axon guidance	1
KCNQ1	971	social behavior	1
PPP3CB	984	regulation of synaptic plasticity	1
GNG4	1039	GABA receptor activation	1
GATA3	1041	sympathetic nervous system development	1
B3GAT1	1061	visual learning	1
APOE	1105	long-term memory; glutamatergic synapse	2
GIPC1	1107	regulation of synaptic plasticity	1

Table S2 (continued).

Genes in the enriched gene sets relating to intelligence, ranking in the top 1500 of the human polarized gene-eigenvector at level 4.

JPH3	1170	exploration behavior; regulation of synaptic plasticity	2
ATXN3	1190	exploration behavior	1
BDNF	1207	axon guidance	1
BAIAP2	1285	axonogenesis; regulation of synaptic plasticity; excitatory synapse; glutamatergic synapse	4
ARRB2	1303	glutamatergic synapse	1
TNR	1353	associative learning	1
HTR1D	1367	regulation of behavior	1
CLSTN3	1375	postsynaptic density membrane	1
RAG1	1410	visual learning	1
ABCA7	1437	visual learning	1
CACNG7	1447	postsynaptic density membrane	1
GRIN2D	1479	regulation of synaptic plasticity; postsynaptic density membrane	2
RIMS2	1497	regulation of synaptic plasticity; positive regulation of inhibitory postsynaptic potential	2

Table S3.
Genes in the enriched gene sets relating to intelligence, ranking in the top 1500 of the human polarized gene-eigenvector at level 9.

Gene Name	Rank	Biological Processes	Process Number
STX3	18	long-term synaptic potentiation	1
PCDH17	22	negative regulation of synaptic transmission; synaptic membrane adhesion	2
RIMS1	25	regulation of synaptic plasticity; positive regulation of inhibitory postsynaptic potential	2
ADGRB1	26	axonogenesis; regulation of synaptic plasticity	2
JPH3	39	regulation of synaptic plasticity	1
ADORA1	45	Sphingolipid signaling pathway	1
PAFAH1B1	74	auditory receptor cell development; cochlea development	2
GNG8	113	GABA receptor activation; GABA B receptor activation	2
DCHS1	142	cochlea development	1
RAPH1	143	axon extension	1
CHRNA9	152	inner ear morphogenesis	1
LRRC4	161	synaptic membrane adhesion; excitatory synapse; Schaffer collateral - CA1 synapse	3
EPHA3	197	axon guidance	1
FYN	205	Schaffer collateral - CA1 synapse; Sphingolipid signaling pathway	2
ADAM23	211	glutamatergic synapse	1
GABRR1	219	GABA receptor activation	1
LMO4	243	motor neuron axon guidance	1
MECP2	251	long-term synaptic potentiation	1
GNAQ	262	Sphingolipid signaling pathway	1
SLC17A7	268	excitatory synapse	1
ADCY1	278	axonogenesis; Schaffer collateral - CA1 synapse; postsynaptic density membrane; glutamatergic synapse; GABA receptor activation; GABA B receptor activation	6
KCNK3	280	cochlea development	1
ARHGAP35	290	axon guidance	1

Table S3 (continued).

Genes in the enriched gene sets relating to intelligence, ranking in the top 1500 of the human polarized gene-eigenvector at level 9.

Gene Name	Rank	Biological Processes	Process Number
KLF7	312	axonogenesis	1
TSHZ3	329	long-term synaptic potentiation	1
RIMS4	364	regulation of synaptic plasticity	1
ARRB2	392	glutamatergic synapse	1
ENAH	408	axon guidance	1
RIMS2	439	regulation of synaptic plasticity; positive regulation of inhibitory postsynaptic potential	2
GABRR2	450	GABA receptor activation	1
PPT1	459	associative learning	1
DSCAM	487	axon guidance	1
NTRK3	488	cochlea development	1
IL1B	511	negative regulation of synaptic transmission	1
DCLK1	515	axon extension	1
CDH6	517	synaptic membrane adhesion	1
SHISA6	544	regulation of postsynaptic neurotransmitter receptor activity	1
SOBP	570	inner ear morphogenesis; cochlea development	2
HOXA2	575	motor neuron axon guidance	1
ADNP	582	negative regulation of synaptic transmission	1
CACNG2	586	Schaffer collateral - CA1 synapse; postsynaptic density membrane	2
NDRG1	588	peripheral nervous system myelin maintenance	1
ADCY9	614	GABA receptor activation; GABA B receptor activation	2
JAG2	616	auditory receptor cell fate commitment	1
SLITRK3	622	synaptic membrane adhesion	1
GIPC1	625	regulation of synaptic plasticity; Schaffer collateral - CA1 synapse	2
PTPRS	638	synaptic membrane adhesion	1

Table S3 (continued).

Genes in the enriched gene sets relating to intelligence, ranking in the top 1500 of the human polarized gene-eigenvector at level 9.

Gene Name	Rank	Biological Processes	Process Number
KRAS	640	Sphingolipid signaling pathway	1
PAK3	705	axonogenesis	1
PVALB	725	cochlea development	1
RPGRIP1L	751	cochlea development	1
DRD2	775	axonogenesis; associative learning	2
CTNNA2	782	axonogenesis	1
JPH4	801	regulation of synaptic plasticity	1
CDK5R1	823	axon guidance	1
EFNB2	888	axon guidance; presynapse assembly; Schaffer collateral - CA1 synapse; postsynaptic density membrane	4
KCNJ15	949	GABA receptor activation; GABA B receptor activation	2
TPH2	976	serotonin biosynthetic process; Serotonin and melatonin biosynthesis	2
IGSF11	994	excitatory synapse	1
SLC17A6	1000	excitatory synapse	1
MAPK9	1039	Sphingolipid signaling pathway	1
ARHGAP22	1063	glutamatergic synapse	1
GRIK1	1082	postsynaptic density membrane	1
CHN1	1108	motor neuron axon guidance	1
DTNBP1	1118	Schaffer collateral - CA1 synapse	1
CASK	1125	Schaffer collateral - CA1 synapse	1
CHD7	1140	inner ear morphogenesis	1
GRIK2	1146	postsynaptic density membrane	1
CDH23	1287	cochlear hair cell ribbon synapse	1
GRM1	1344	Schaffer collateral - CA1 synapse; postsynaptic density membrane	2
HTR3B	1357	serotonin receptor signaling pathway; serotonin-activated cation-selective channel complex; serotonin-gated cation-selective channel activity	3

Table S3 (continued).

Genes in the enriched gene sets relating to intelligence, ranking in the top 1500 of the human polarized gene-eigenvector at level 9.

Gene Name	Rank	Biological Processes	Process Number
POU4F2	1389	axon extension	1
AGT	1417	associative learning	1
ASAP1	1441	glutamatergic synapse	1
GABBR2	1452	GABA receptor activation; GABA B receptor activation	2
APBB1	1467	axonogenesis	1
TSC1	1497	associative learning	1

Table S4.
High-ranking cognition-related genes standing around the positive pole of the human 4th and 9th motif-eigenvectors.

	**Gene Name**	**Description**	**Rank in human**	**Rank in bonobo**
**Level 4+**	DNMT3L	related to cognitive decline	6	9957
	NAE1	differential expression in the hippocampus	28	14175
	SHANK1	pivotal for cognition and synaptic structure; expressed in glial cells	91	12897
	ZBTB45	involved in glial differentiation of oligodendrocyte progenitor and neural cells	85	15330
**Level 9+**	STX3	related to memory	18	2221
	IFNAR1	receptor of type I interferon, important for memory and cognition	34	3028
	N4BP1	involved in neural stem cell differentiation	20	9908
	MED15	significantly expressed in the nervous system	76	1872
	KLHL22	crucial for maintaining and differentiating the precursor pool	100	2523
	PLOD2	correlated with brain arteriovenous malformations	30	17157
	VRK2	related to schizophrenia (SCZ) and major depressive disorder (MDD)	48	15105
	B4GALT5	involved in neuronal generation and myelin formation	119	4605
